# Acetoacetate protects macrophages from lactic acidosis-induced mitochondrial dysfunction by metabolic reprograming

**DOI:** 10.1038/s41467-021-27426-x

**Published:** 2021-12-08

**Authors:** Clément Adam, Léa Paolini, Naïg Gueguen, Guillaume Mabilleau, Laurence Preisser, Simon Blanchard, Pascale Pignon, Florence Manero, Morgane Le Mao, Alain Morel, Pascal Reynier, Céline Beauvillain, Yves Delneste, Vincent Procaccio, Pascale Jeannin

**Affiliations:** 1 Univ Angers, Université de Nantes, INSERM, CRCINA, LabEx IGO, SFR ICAT, F-49000 Angers, France; 2 Univ Angers, CHU d’Angers, INSERM, CNRS, MitoVasc, SFR ICAT, F-49000 Angers, France; 3 Département de Biochimie et Génétique, CHU d’Angers, Angers, France; 4 GEROM, Université d’Angers, Angers, France; 5 Département de Pathologie Cellulaire et Tissulaire, CHU d’Angers, Angers, France; 6 Laboratoire d’Immunologie et Allergologie, CHU d’Angers, Angers, France; 7 Univ Angers, SFR ICAT, SCIAM, F-49000 Angers, France; 8 Institut de Cancérologie de l’Ouest, F-49000 Angers, France; 9 Laboratoire de Biochimie et biologie moléculaire, CHU d’Angers, Angers, France

**Keywords:** Monocytes and macrophages, Cancer microenvironment, Mitophagy, Energy metabolism

## Abstract

Lactic acidosis, the extracellular accumulation of lactate and protons, is a consequence of increased glycolysis triggered by insufficient oxygen supply to tissues. Macrophages are able to differentiate from monocytes under such acidotic conditions, and remain active in order to resolve the underlying injury. Here we show that, in lactic acidosis, human monocytes differentiating into macrophages are characterized by depolarized mitochondria, transient reduction of mitochondrial mass due to mitophagy, and a significant decrease in nutrient absorption. These metabolic changes, resembling pseudostarvation, result from the low extracellular pH rather than from the lactosis component, and render these cells dependent on autophagy for survival. Meanwhile, acetoacetate, a natural metabolite produced by the liver, is utilized by monocytes/macrophages as an alternative fuel to mitigate lactic acidosis-induced pseudostarvation, as evidenced by retained mitochondrial integrity and function, retained nutrient uptake, and survival without the need of autophagy. Our results thus show that acetoacetate may increase tissue tolerance to sustained lactic acidosis.

## Abstract (web summary)

Lactic acidosis is a metabolic state that occurs in injured tissues. Here the authors show that macrophages, in order to remain functional in acidosis, reduce their mitochondrial mass by mitophagy and rely on autophagy for survival, with mitochondrial integrity retained using acetoacetate as alternative fuel.

## Introduction

Normal tissues have an extracellular pH (pHe) of ≈7.4. Interstitial acidification (with pHe down to 6.5) is usually observed in inflammatory processes associated to solid tumors, and infections^1–4^. Indeed, rapid cell proliferation and/or hypoxia lead to an increase in glycolysis, with the release of lactic acid (LA), which dissociates, in the extracellular space, into lactate and protons, the excess of protons causing extracellular acidosis^5,6^. Extracellular lactate concentrations, which range between 1.8 and 2 mM in resting tissues, can reach up to 20 mM in wounds, and 40 mM in solid tumors^6^. Cells tightly control their intracellular pH, but prolonged extracellular acidosis can affect several aspects of cellular homeostasis, including metabolism, signaling, and transcriptional activities^7–9^.

Macrophages are innate myeloid cells found in almost all tissues. They play a key role in maintaining tissue homeostasis, promoting tissue repair and protective anti-infectious immunity, and in controlling tumor development^10^. Macrophages are sometimes likened to “firefighters”, due to the role they play in damaged and infected areas. Through their functional plasticity, macrophages continuously adapt their phenotypes to signals (such as immune mediators or metabolites) present in the microenvironment, thereby responding precisely to the tissue needs^10–12^.

In the event of severe inflammation/injury, the resident macrophages may be overwhelmed, and monocytes are therefore recruited and differentiated into macrophages^10^. The differentiation of monocytes into macrophages probably generally occurs in damaged tissues, in which LA levels may be extremely high, resulting in a low pHe. However, the differentiation of human macrophages has mostly been studied at physiological pH (pHe = 7.2-7.4), which seems to be of limited relevance given that monocytes are mostly recruited to damaged sites. Human macrophages are long-lived cells and their proliferation capacity is unclear^13,14^. The ability of monocytes to survive and differentiate into macrophages under lactic acidosis conditions may therefore be crucial to ensure the maintenance of a pool of functional macrophages in damaged tissues. We and others have recently reported that LA modulates the functional phenotype of murine and human macrophages^15,16^. However, the mechanisms by which monocytes/macrophages survive in prolonged lactic acidosis conditions have yet to be determined. By contrast, the metabolic adaptations by which tumor cells survive and proliferate under lactic acidosis conditions have been studied in detail^4,17–20^.

The objective of this study was to investigate how human monocytes survive and differentiate into macrophages under lactic acidosis conditions. Experiments were conducted under atmospheric oxygen and in the presence of glucose, to rule out the effect of oxygen or glucose deprivation.

Here we show that (i) extracellular acidosis induces mitochondrial membrane depolarization and decreases nutrient consumption, resulting in dependence of the macrophages on a transient phase of autophagy for survival, and (ii) the ketone body acetoacetate protects the mitochondria from acidosis-induced depolarization and mitophagy, allowing the cells to continue to metabolize nutrients, thereby avoiding the need for self-catabolism to survive.

## Results

### Human macrophages display a reduced mitochondrial mass in lactic acidosis

We investigated the mechanisms by which human monocytes cope with LA by culturing monocytes with GM-CSF (Mφ) without or with 10 mM LA (LA-Mφ; pH = 6.5), the maximum non-toxic concentration for Mφ (Supplementary Fig. 1). This concentration of LA and a pHe of 6.5 are commonly observed in inflamed tissues^1,6^.

**Fig. 1:**
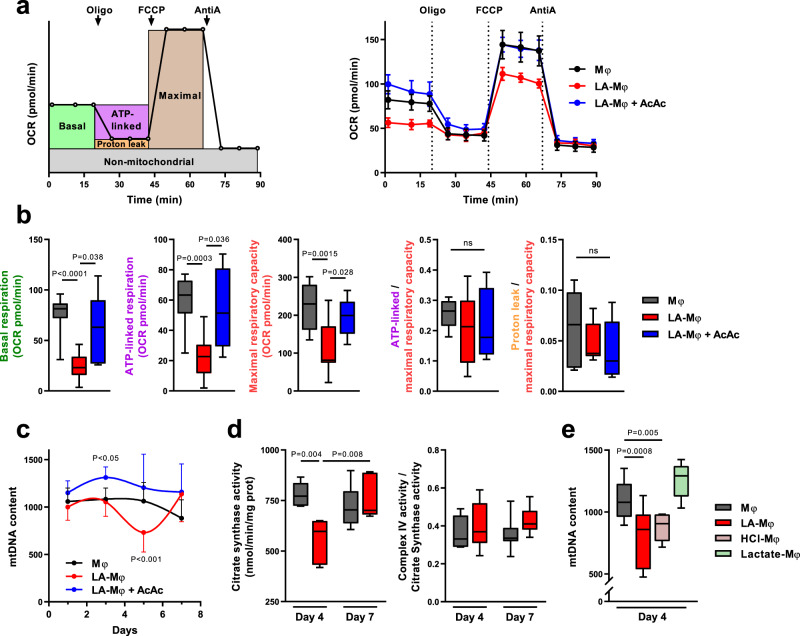
Sustained acidosis affects macrophage mitochondrial mass. **a**–**e** Monocytes were polarized into macrophages in the absence (Mφ) or presence of lactic acid (LA-Mφ), lactic acid and acetoacetate (LA-Mφ + AcAc), sodium lactate (Lactate-Mφ), or under acidosis (HCl-Mφ). **a**, **b** Monitoring of oxygen consumption rate (OCR) in day 4 macrophages following a sequential addition of oligomycin (oligo), carbonyl cyanide p-(trifluoromethoxy) phenylhydrazone (FCCP), and antimycin A (AntiA). Basal OCR, ATP-linked respiration, maximal respiratory capacity, and proton-leak respiration were determined as described in the Methods section. **a** Schematic (left panel) and representative (right panel) OCR profiles (mean ± SD; *n* = 8). **b** Metabolic parameters obtained from OCR profiling. ATP-linked respiration/maximal respiratory capacity (ATP-linked/maximal) and proton leak/maximal respiratory capacity (proton leak/maximal) ratios were calculated (*n* = 11 for Mφ and LA-Mφ; *n* = 5 for LA-Mφ + AcAc). **c** Quantification of mitochondrial DNA (mtDNA) copy number by qPCR from day 1 to day 7 (mean ± SD; *n* = 10). Two-way ANOVA followed by a Tukey post hoc test was performed for statistical analysis. **d** Citrate synthase activity and complex IV/citrate synthase ratio at day 4 and 7 (*n* = 6). **e** mtDNA was quantified on day 4 (*n* = 12 for Mφ and LA-Mφ; *n* = 6 for HCl-Mφ and Lactate-Mφ). **a**–**e** The boxplots display a median line, interquartile range (IQR) boxes, min to max whiskers; two-tailed Mann–Whitney *U* test was performed for statistical analysis unless specified otherwise; ns not significant. Source data are provided in a Source Data file.

We first compared the mitochondrial respiration rates of macrophages, by measuring the oxygen consumption rate (OCR) of day 4 Mφ and LA-Mφ (Fig. 1a). Basal respiration, ATP-linked respiration, and maximum respiratory capacity were significantly lower in LA-Mφ than in Mφ (Fig. 1a, b). This global decrease in all the respiration rates suggests that mitochondrial mass was diminished on day 4 LA-Mφ. Consistent with this hypothesis, mitochondrial DNA (mtDNA) copy number, an indicator of the mitochondrial mass, decreased markedly from day 3 to day 5 in LA-Mφ, subsequently displaying a trend towards normalization at day 7 (Fig. 1c). Accordingly, the activity of citrate synthase, another marker of mitochondrial mass, was also reduced in day 4 LA-Mφ compared to Mφ (Fig. 1d). By contrast, the residual mitochondria maintain a quite functional electron transport chain (ETC), as the part of the maximal capacity used for sustaining ATP synthesis is not significantly decreased in LA-Mφ compared to Mφ (Fig. 1b) and the complex IV activity normalized to citrate synthase activity was not affected (Fig. 1d).

Lactic acidosis results from the extracellular accumulation of lactate and protons. We, therefore, investigated whether the decrease in mitochondrial mass observed in LA-Mφ (10 mM LA, pH = 6.5) was due to acidosis or lactosis. We determined the mtDNA content of macrophages generated under conditions of acidosis (HCl-Mφ; 10 mM HCl, pH = 6.5) or lactosis (lactate-Mφ; 10 mM sodium lactate, pH = 7.3) alone. We found that the mtDNA copy number was significantly reduced in macrophages under acidosis, but not under lactosis conditions (Fig. 1e). In subsequent experiments, to mimic the pathophysiological situations, we have generated macrophages under lactic acidosis. We found that monocytes/macrophages undergo a transient decrease in mitochondrial mass under conditions of acidosis.

### The expression of genes involved in mitochondrial biogenesis is unaffected by lactic acidosis

Mitochondria undergo constant remodeling to maintain correct function. A decrease in mitochondrial mass may therefore result from either a reduction of mitochondrial biogenesis and/or an increase in mitophagy^21,22^. We investigated the process affected by LA, by first analyzing the impact of LA on the expression of genes involved in mitochondrial biogenesis. The expression of *TFAM*, *NRF2*, *PGC-1β* did not significantly differ between LA-Mφ and Mφ, whatever the time-point analyzed, and *PGC-1α* was poorly expressed (Supplementary Fig. 2). These results suggest that the decrease in mitochondrial mass in LA-Mφ does not result from a reduction in mitochondrial biogenesis.

### The autophagic flux in macrophages is enhanced during lactic acidosis

Mitochondrial content is coordinately regulated by both mitochondrial biogenesis and mitochondrial selective autophagy (mitophagy), in response to cellular metabolic state and stress. Mitophagy is the selective removal of dysfunctional mitochondria by the autophagic machinery. More precisely, dysfunctional mitochondria are engulfed in autophagosomes, which then fuse with lysosomes, leading to the degradation of the content of the resulting phagolysosomes. Since mitochondrial biogenesis was not downregulated, we hypothesized that autophagic activity increases in macrophages during lactic acidosis.

**Fig. 2:**
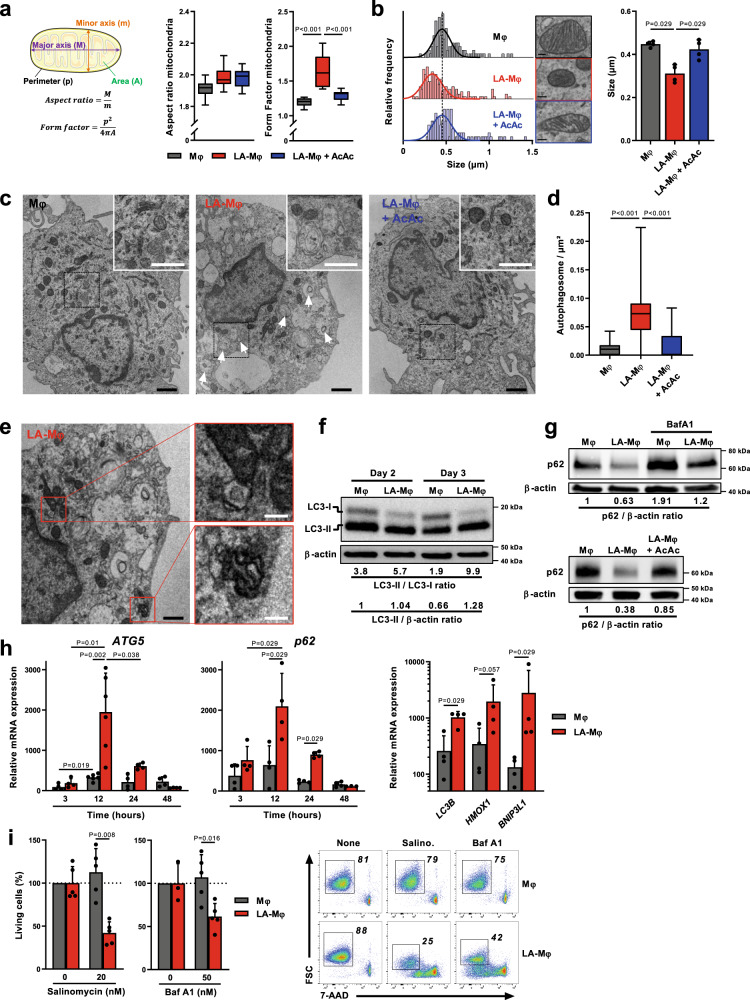
Human macrophages display upregulation of autophagic flux during lactic acidosis. **a**–**i** Monocytes were polarized without lactic acid (Mφ), with lactic acid (LA-Mφ) or with lactic acid and acetoacetate (LA-Mφ + AcAc). **a** Schematic representation of the morphological meaning of aspect ratio and form factor indexes (left) and analyses of these indexes by confocal microscopy in macrophages at day 5 (right) (*n* = 10 cells examined over 2 independent replicates). **b** Size of mitochondria measured on TEM micrographs at day 3. Left panel, representative frequency histograms (size bar, 0.1 µm); right panel, a compilation of the results (mean ± SD; *n* = 100–150 mitochondria examined over 4 independent replicates). **c** Representative TEM micrographs of the indicated Mφ subsets on day 3 (representative images shown from three independent biological experiments); arrows, autophagosomes; black size bar, 1 µm; white size bar (insert), 0.3 µm. **d** Quantification of autophagic vesicles per cell on day 3 (*n* = 30 cells examined over 3 biological replicates). **e** Representative TEM micrographs of mitophagy vacuoles in day 3 LA-Mφ (representative images shown from three independent biological experiments); black size bar, 1 µm; white size bar (insert), 0.3 µm. **f**, **g** Western blotting analysis of LC3-I, LC3-II (f), p62 (g), and β-actin (**f**–**g**) in day 3 Mφ, in the presence or absence of bafilomycin A1 (BafA1) or AcAc. The LC3-II/LC3-I, LC3-II/β-actin, and p62/β-actin band intensity ratios are indicated (representative images shown from three independent experiments). **h** Levels of *ATG5*, *p62*, *LC3B*, *HMOX1*, and *BNIP3L1* mRNA were assessed by RT-qPCR at the indicated time points (left and middle panels) and at 12 h (right panel) and were normalized to the expression of the *RPS18* housekeeping gene (*n* = 4). **i** Day 2 Mφ was exposed to salinomycin or BafA1 for 24 h, and cell viability was evaluated by flow cytometry with 7-AAD staining (*n* = 5, left panel). Results are expressed as percent viability compared to the Mφ condition, which has been assigned a value of 100%. Representative dot plots showing 7-AAD staining versus FSC (right panel). **a**–**i** The boxplots display a median line, interquartile range (IQR) boxes, min to max whiskers; a two-tailed Mann–Whitney *U* test was performed for statistical analysis. Source data are provided in a Source Data file.

Fission events, which segregate dysfunctional mitochondria and generate smaller mitochondria, are a prerequisite for the elimination of dysfunctional mitochondria by mitophagy^23^. Confocal microscopy revealed changes in the organization of the mitochondrial network architecture in LA-Mφ relative to Mφ, with higher values for the morphological form factor (Fig. 2a). However, the mitochondria in Mφ were almost dot-like (form factor values close to 1) (Fig. 2a). As such, mitochondrial budding during fission would result in higher form factor values that could be misinterpreted as a filamentous network. Transmission electron microscopy (TEM) revealed that mean mitochondrial size was smaller in LA-Mφ than in Mφ, with a shift in the entire distribution towards smaller sizes (Fig. 2b). These modifications of the mitochondrial network, at the micro- and nanoscale, are entirely consistent with a more fragmented mitochondrial network and mitophagy^24^. The number of autophagosomes in the cytoplasm of Mφ and LA-Mφ was determined by TEM, a standard method for monitoring autophagy and mitophagy^25^. On day 3, LA-Mφ contained larger numbers of autophagosomes than Mφ (Fig. 2c, d) and figures of mitophagy have been observed only in LA-Mφ (Fig. 2e). To confirm the enhanced autophagic activity in LA-Mφ, the levels of the autophagosome-associated protein LC3-II were analyzed in the presence of bafilomycin A1 (BafA1), an inhibitor of the vacuolar H^+^ ATPase that prevents lysosomal acidification and interferes with autophagosome/LC3-II degradation^26^. We found that LC3-II/LC3-I and LC3-II/β-actin ratios were higher in LA-Mφ than in Mφ at day 2 and 3 (Fig. 2f), confirming the presence of a large number of mature autophagosomes in LA-Mφ. The expression of p62, which binds directly to LC3 and is degraded by autophagy, also decreased strongly in LA-Mφ, and this reduction was counterbalanced to some extent by BafA1, demonstrating that the autophagic flux was still functional in LA-Mφ (Fig. 2g, upper panel). In addition, consistent with enhanced autophagic activity, levels of the mRNAs encoding *ATG5*, *p62*, *LC3B*, *HMOX1*, and *BNIP3L1*, all involved in autophagy^27^, were transiently upregulated in LA-Mφ relative to Mφ, peaking at 12 h (Fig. 2h). These observations ruled out the possibility of the increase in autophagosome numbers in LA-Mφ being due to accumulation as a result of defective elimination. As previously reported^28^, monocytes differentiated in the presence of GM-CSF displayed a discrete increase in *ATG5* and *p62* mRNA levels (Fig. 2h). In conclusion, autophagic flux is functional and exacerbated in macrophages under lactic acidosis conditions.

### Macrophages rely on autophagy to survive during lactic acidosis

We, therefore, hypothesized that monocytes/macrophages use autophagy to survive prolonged exposure to lactic acidosis. To test this hypothesis, we used salinomycin and BafA1, two autophagy inhibitors that have been shown to be active under acidic conditions^29–31^. The addition of salinomycin or BafA1 to day 3 LA-Mφ significantly decreased their survival (Fig. 2i and Supplementary Fig. 3a), demonstrating that Mφ relies on autophagy to survive lactic acidosis. Autophagy, therefore, appears to be an adaptive protective response enabling macrophages to survive in a lactic acidosis microenvironment.

### Prolonged lactic acidosis induces mitochondrial depolarization in macrophages, resulting in energetic stress

Self-catabolic autophagy is strongly induced in response to nutrient starvation, to provide energy substrates; cellular material is, therefore sacrificed, to allow ATP production^32,33^. In lactic acidosis, monocytes/macrophages are dependent on autophagic processes to survive. We, therefore, hypothesized that cells in lactic acidosis undergo energetic and metabolic stress to which they must adapt in order to cope with “starvation”, despite the presence of oxygen and nutrients.

**Fig. 3:**
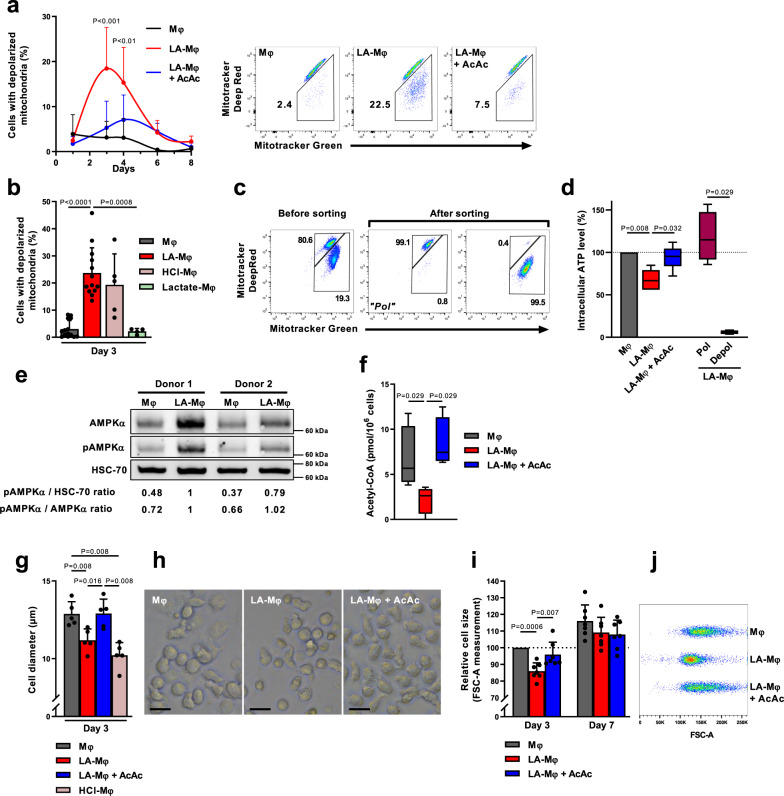
Macrophages in conditions of lactic acidosis display mitochondrial depolarization and characteristics typical of starving cells. **a–k** Monocytes were polarized into macrophages in the absence (Mφ) or presence of lactic acid (LA-Mφ), lactic acid and acetoacetate (LA-Mφ + AcAc), sodium lactate (Lactate-Mφ), or under acidosis (HCl-Mφ). **a**, **b** Cells with depolarized mitochondrial membrane potential (ΔΨm) were analyzed by flow cytometry using MitoTracker Green and MitoTracker Deep Red probes at the indicated time point (**a**) (mean ± SD; *n* = 4, two-way ANOVA followed by a Tukey post hoc test was performed for statistical analysis) or at day 3 (**b**) (mean ± SD; *n* = 14 for Mφ; *n* = 13 for LA-Mφ; *n* = 5 for HCl-Mφ; *n* = 4 for Lactate-Mφ). Representative dot plots on day 3 (**a**, right panel). **c** Macrophages with or without depolarized mitochondrial membrane potential (“Depol” and “Pol” populations, respectively) were isolated from day 3 LA-Mφ by flow cytometry with MitoTracker Green and MitoTracker Deep Red probes. Purity was >99%. **d** Intracellular ATP levels were measured in a semiquantitative assay. The results were normalized against the Mφ condition for each donor (*n* = 5). **e** Levels of total AMPKα, AMPKα phosphorylated on Thr172 (pAMPKα) and HSC-70 were analyzed by Western blotting at 6 h (representative images from four independent experiments); the values correspond to pAMPKα/HSC-70 and pAMPKα/AMPKα band intensity ratios. **f** Intracellular acetyl-CoA levels determined on day 3 (*n* = 4). **g**–**j** Cell size was measured on day 3 (**g**–**j**) and day 7 (**i**), by determining the mean ± SD of the diameter of Mφ in each population (**g**, *n* = 5) or relative cell size by flow cytometry with the FSC-A parameter (**i**, *n* = 7). **h** Photomicrographs of the Mφ population on day 3 (representative images from five independent experiments); size bar, 10 µm. **j** Representative dot plots from one donor of FSC-A measurement. **a**–**j** The boxplots display a median line, interquartile range (IQR) boxes, min to max whiskers; a two-tailed Mann–Whitney *U* test was performed for statistical analysis unless specified otherwise. Source data are provided in a Source Data file.

Mitochondrial depolarization to a dysfunctional level below a certain membrane potential (Δψm) is a prerequisite for mitophagy^24^. We, therefore, evaluated the presence of depolarized mitochondria, i.e., displaying a loss of Δψm in Mφ and LA-Mφ. We distinguished between total mitochondria and depolarized mitochondria, through a combination of staining with MitoTracker Green (total Δψm-independent mitochondrial content dye) and MitoTracker Deep Red (Δψm-dependent mitochondrial dye, suitable for use in an acidic medium)^34–36^. We observed a massive accumulation of depolarized mitochondria (MitoTracker Green^high^ MitoTracker Deep Red^low^) in LA-Mφ, peaking at day 3 (Fig. 3a and Supplementary Fig. 3b) and in HCl-Mφ but not in lactate-Mφ (Fig. 3b and Supplementary Fig. 3b). In parallel, after three days of lactic acidosis, macrophages displayed a decrease in cellular ATP content due a fall in ATP levels in the fraction of cells with depolarized mitochondria (Fig. 3c, d and Supplementary Fig. 3b). The decrease in mitochondrial membrane potential, therefore, impairs oxidative ATP production, leading to energy stress.

### Macrophages display metabolic and cellular changes typical of starving cells during lactic acidosis

Decreased cellular ATP content could activate AMPK, thereby promoting autophagy^37,38^. LA-Mφ displayed an early increase in AMPK Thr172 phosphorylation (Fig. 3e). Lactic acidosis also upregulated AMPK expression in macrophages (Fig. 3e), as reported for melanoma cells under similar conditions^18^. A decrease in nutrient supply toward the respiratory chain would compromise the mitochondrial capacity to maintain mitochondrial potential and ATP synthesis. We thus analyzed the impact of lactic acidosis on intracellular levels of acetyl-coenzyme A (AcCoA), a major integrator of nutritional status at the crossroads of fat, carbohydrate, and protein catabolism^39^. A decrease in intracellular AcCoA levels induced by nutrient deficiency triggers autophagy^39,40^. We observed that AcCoA levels were significantly lower in LA-Mφ than in Mφ (Fig. 3f). Thus, myeloid cells subjected to lactic acidosis have characteristics similar to those of starving cells.

Finally, LA-Mφ (Fig. 3g–j) and HCl-Mφ (Fig. 3g) cells were significantly smaller than Mφ, with the smallest cell sizes recorded on day 3 and a trend towards cell size normalization by day 7 (Fig. 3I, j and Supplementary Fig. 3c). Size reduction of cells is generally considered to be a biophysical change occurring under stress conditions, such as nutrient deprivation, enabling cells to minimize energy demand and expenditure^41^. Surprisingly, such changes have rarely been associated with autophagy^42^.

### Lactic acidosis greatly decreases nutrient catabolism

**Fig. 4:**
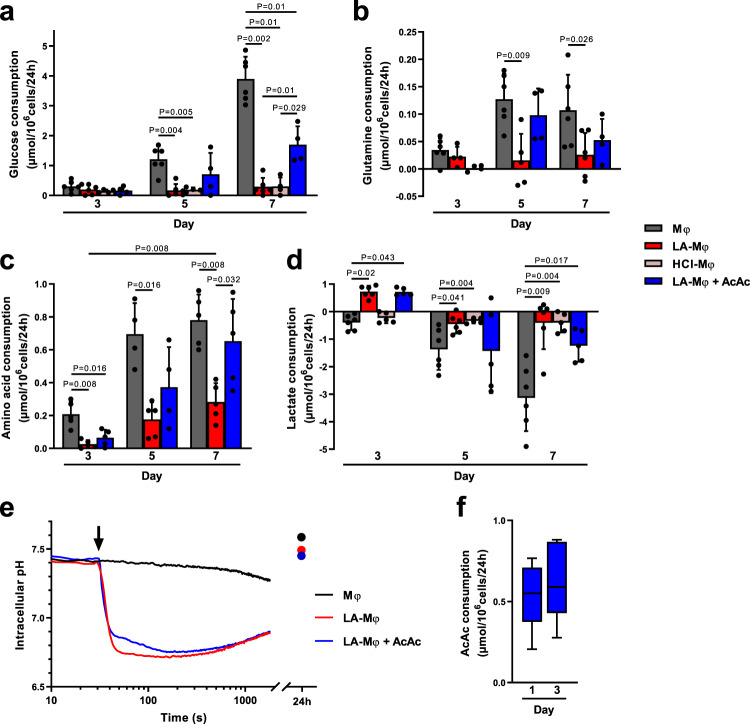
Lactic acidosis is associated with pseudostarvation. **a**–**e** Monocytes were polarized into macrophages in the absence (Mφ) or presence of lactic acid (LA-Mφ), lactic acid and acetoacetate (LA-Mφ + AcAc), or under acidosis (HCl-Mφ). Glucose (**a**), glutamine (**b**), free l-amino acids (**c**), and lactate (**d**) were quantified at days 3, 5, and 7 in cell culture supernatants of Mφ, LA-Mφ, LA-Mφ + AcAc, and HCl-Mφ. Results are expressed in µmol/10^6^ cells/24 h, with positive values for consumption and negative values for production (mean ± SD; Mφ and LA-Mφ: *n* = 6; HCl-Mφ: *n* = 4 (**a**) or *n* = 5 (**d**); LA-Mφ + AcAc: *n* = 4 (**a**, **b**) or *n* = 5 (**c**, **d**)). **e** Intracellular pH was measured by flow cytometry with the SNARF probe. Monocytes were loaded with the SNARF probe and analyzed for 30 s before the addition (arrow) of 10 mM lactic acid (LA-Mφ), with or without 5 mM acetoacetate (LA-Mφ + AcAc). The acquisition was then prolonged for an additional 30 min. Probe loading and acquisition were repeated at 24 h. Representative results from one of three independent experiments are shown. **f** Acetoacetate (AcAc) was quantified on days 1 and 3 in LA-Mφ + AcAc culture supernatants (*n* = 5). Results are expressed in µmol/10^6^cells/24 h. **a**–**f** The boxplots display a median line, interquartile range (IQR) boxes, min to max whiskers; a two-tailed Mann–Whitney *U* test was performed for statistical analysis. Source data are provided in a Source Data file.

We investigated the mechanism underlying the decrease in AcCoA levels and the pseudostarvation in LA-Mφ, by comparing the ability of monocytes/macrophages to consume nutrients in the presence and absence of LA. Glucose, lactate, and amino acids were quantified in cell culture supernatants during differentiation. In LA-Mφ, glucose consumption was almost undetectable in the first few days and the uptake of amino acids, including glutamine, in particular, decreased strongly, consistent with a general limitation of substrate supplies and a cellular starvation state. Amino-acid uptake was recommenced on day 5 (Fig. 4b, c), but glucose consumption did not (Fig. 4a). Thus, under acidosis, monocytes treated with LA or HCl had low rates of glycolysis. As previously observed^16,20,43^, monocytes treated with LA or HCl lost their capacity to use glucose early in differentiation, despite cell–surface expression of the glucose transporters GLUT1, GLUT3, and GLUT5 (Supplementary Fig. 4). This defect was maintained until day 7 (Fig. 4a, d) and was associated with an extracellular pH (pHe) that remained stable over time (Supplementary Fig. 5). In Mφ exposed to LA, extracellular lactate concentrations, which reflect the balance between LA uptake and production, showed consumption of lactate on the third day in LA-Mφ (Fig. 4d)^15,16^. This result suggests that cellular lactate oxidation was occurring, but this metabolic adaptation did supply sufficient amounts of the substrate to the respiratory chain to support further ATP synthesis, as demonstrated by the lower AcCoA levels (Fig. 3f), mitochondrial depolarization (Fig. 3a and supplementary Fig. 3b), and decrease in ATP content (Fig. 3d and Supplementary Fig. 3b).

A low extracellular pH (pHe) has been shown to lead to acidification of the cytosolic compartment of tumor cells^18,44^, thereby inhibiting glycolysis^19,20^. Indeed, changes in intracellular pH (pHi) affect intracellular gradients of protons and membrane symporters and may modify the activities of many enzymes^45,46^. We, therefore, evaluated the impact of extracellular acidosis on the pHi of monocytes (Fig. 4e). The pHi of monocytes exposed to a pHe of 7.4 remained stable over time, but the exposure of monocytes to a pHe of 6.5 led to a sharp drop in pHi, followed by a return to normal values on day 1 (Fig. 4e). Thus, under lactic acidosis conditions, monocytes/macrophages display a strong but transient reduction of pHi. In conclusion, these observations indicate that, in an acidic environment, monocytes/macrophages reduce their nutrient consumption, entering a state of pseudostarvation in which they rely on self-catabolism to survive.

### The metabolic stress induced by lactic acidosis compromises macrophage functions

**Fig. 5:**
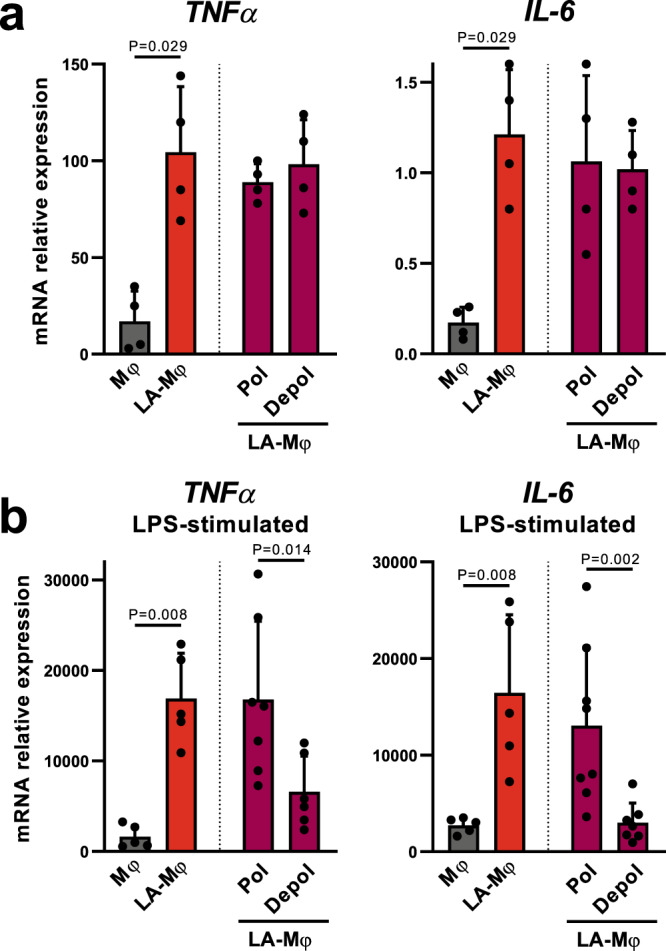
Relative expression of cytokine mRNAs by LA-Mφ with depolarized mitochondria. **a**, **b** Monocytes were polarized into macrophages by incubation in the absence (Mφ) or presence of lactic acid (LA-Mφ) for 3 days. LA-Mφ with and without depolarized mitochondrial membranes (“Depol” and “Pol” populations, respectively) were sorted by flow cytometry with MitoTracker probes. Cells were unstimulated (**a**) or stimulated with LPS for 3 h (**b**) and the levels of *TNFα* and *IL-6* mRNA were assessed by RT-qPCR. The results were expressed as mRNA levels relative to those for the housekeeping gene *RPS18* (mean ± SD, **a** *n* = 4; **b** *n* = 5 for Mφ and LA-Mφ; *n* = 7 for “Depol” and “Pol” populations). A two-tailed Mann–Whitney *U* test was performed for statistical analysis. Source data are provided in a Source Data file.

We then investigated the possible effect of the metabolic and energetic stress of macrophages exposed to lactic acidosis on their function. As we had already shown that human LA-Mφ have an inflammatory phenotype^16^, we compared the levels of *IL-6* and *TNFα* mRNAs in LA-Mφ with and without depolarized mitochondria. In the absence of stimulation, FACS-sorted day 3 LA-Mφ with and without depolarized mitochondria had similar levels of *IL-6* and *TNFα* mRNA, higher in both cases than those in Mφ not exposed to lactic acidosis (Fig. 5a). Both subsets of LA-Mφ also expressed similar levels of the cell surface molecules CD86 and MHC II (Supplementary Fig. 6) and no endoplasmic reticulum stress was detected by TEM in LA-Mφ. Human macrophages only produce cytokines if they are stimulated^47^. In response to LPS stimulation, LA-Mφ with depolarized mitochondria produced significantly smaller amounts of *IL-6* and *TNFα* mRNA than Mφ without depolarized mitochondria (Fig. 5b). Thus, monocytes/macrophages under lactic acidosis display metabolic and morphological changes typical of starving cells, despite the presence of oxygen and nutrients, in a process called pseudostarvation, and this energy crisis compromises their functions in response to stimulation.

### AcAc prevents lactic acidosis-induced pseudostarvation and mitophagy

In fasting periods, in which carbohydrate availability is reduced, the ketone bodies AcAc and β-OHB are de novo-synthesized within hepatocyte mitochondria, via fatty acid β-oxidation. They serve as vital alternative metabolic fuel sources for extrahepatic cells^48^. Mitochondria from many cells, including murine macrophages, and tissues (except liver), have been shown to oxidize AcAc^48,49^. As reported for murine macrophages^49^, we first confirmed that human monocytes express the mRNA encoding the two enzymes *OXCT1* and *ACAT1* involved in AcAc oxidation (Supplementary Fig. 7a). Mitochondrial ketone body catabolism generates succinate and acetyl-CoA that can be terminally oxidized within the TCA cycle, thereby directly supplying substrates to the respiratory chain through reactions that do not require ATP^48^. As a consequence, macrophages generated in AcAc exhibited a significantly enhanced oxidative metabolism (with enhanced routine respiration, ATP-linked respiration, and ATP-linked/maximal respiration) while the respiratory chain capacity remains unchanged (Supplementary Fig. 8a). It is worth noting that AcAc alone does not affect mitochondrial depolarization (Supplementary Fig. 8b).

Therefore, we then investigated the possible use of ketone bodies by monocytes/macrophages, as an alternative fuel to bypass lactic acidosis-induced pseudostarvation. Monocytes were differentiated into macrophages under lactic acidosis conditions, in the presence or absence of 5 mM AcAc (LA-Mφ + AcAc). Surprisingly, despite the presence of LA, LA-Mφ + AcAc did not exhibit the mitochondrial depolarization observed in the presence of LA alone (Fig. 3a and supplementary Fig. 3b). Basal respiration and ATP-linked respiration, but also maximal respiratory chain capacity were significantly upper in LA-Mφ + AcAc compared to LA-Mφ (Fig. 1a, b) and, correspondingly, the cellular ATP content was also restored (Fig. 3d). This protective effect was observed with both lithium AcAc and sodium AcAc but not with chloride lithium used as a negative control (Supplementary Fig. 9).

In parallel, AcAc protected the mitochondrial network architecture, as shown by the similarity of mitochondrial morphology and size between these cells and Mφ (Fig. 2a, b). Moreover, LA-Mφ + AcAc displayed no reduction of the mitochondrial mass, which instead even increased slightly by day 2 (Fig. 1c), accompanied by an expression of *NRF2* that tends to increase relative to LA-Mφ (Supplementary Fig. 2). Autophagy assessed by TEM (Fig. 2c, d) or molecularly (Fig. 2g, lower panel) was not upregulated in day 3 LA-Mφ + AcAc relative to Mφ. LA-Mφ may no longer need to use autophagy in the presence of AcAc, as they not only consume AcAc (Fig. 4f), but also retain a partial capacity to take up glucose and amino acids and to produce lactate (Fig. 4a–d). AcAc metabolization was necessary to prevent acidosis-induced mitophagy, as two inhibitors of the mitochondrial thiolase ACAT1, the enzyme which oxidizes AcAc to AcetylCoA, reduced the protective effect of AcAc on LA-induced mitochondrial depolarization (Supplementary Fig. 10). Accordingly, AcAc prevented the reduction of intracellular AcCoA levels (Fig. 3f) and of cell size (Fig. 3g–j) induced by LA. Finally, the immediate but short-term decrease in pHi induced by extracellular acidosis remained detectable in the presence of AcAc (Fig. 4e).

**Fig. 6:**
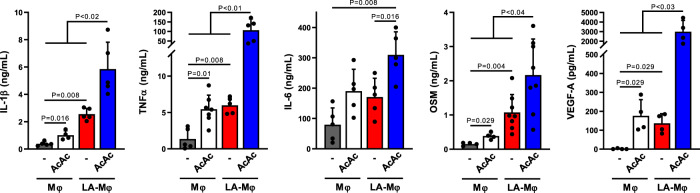
Impact of AcAc on cytokine secretion by LA-Mφ. Monocytes were differentiated into macrophages in the absence (Mφ) or presence of lactic acid (LA-Mφ), with or without acetoacetate (AcAc). On day 5, cells were stimulated for 16 h with LPS and IL-1β, TNFα, IL-6, oncostatin M (OSM), and VEGF-A were quantified by ELISA in the supernatants (mean ± SD, *n* = 4–8). A two-tailed Mann–Whitney *U* test was performed for statistical analysis. Source data are provided in a Source Data file.

As AcAc protects macrophage energetic functions in case of acidosis, we then analyzed its impact on the phenotype of human LA-Mφ. AcAc has been previously reported to enhance IL-6 and TNFα production by U937 cell line^50,51^ and to modulate murine macrophage properties^49^. We and others have shown that LA-Mφ exhibits an inflammatory phenotype and produces growth factors (OSM^high^ and VEGF^high^). Monocytes differentiated in the presence of AcAc exhibited a TNFα^high^ IL-1β^high^ IL-6^high^ OSM^high^ and VEGF^high^ phenotype, as macrophages differentiated in lactic acid (Fig. 6). That these two substrates fuel mitochondria in myeloid cells^15,49^ may help explain why they imprint a similar cytokine signature. AcAc plus LA resulted in an upregulation of cytokine secretion by macrophages (Fig. 6), showing that AcAc not only bypasses LA-induced macrophage starvation and autophagy but also improves their ability to produce cytokines.

Finally, consistent with the observation that murine macrophages do not metabolize β-OHB and do not express BDH1, the mitochondrial enzyme that oxidizes β-OHB to AcAc^49^, BDH1 protein was undetectable in human monocytes (Supplementary Fig. 7b) and β-OHB failed to protect monocytes/macrophages from acidosis-induced mitochondrial depolarization (Supplementary Fig. 9).

Thus, AcAc appears to act as an alternative fuel source avoiding the pseudostarvation state during lactic acidosis, thereby maintaining mitochondrial integrity, and preventing cellular dysfunction due to acidic stress, which could finally increase tissue resistance to acidosis.

## Discussion

Lactic acidosis is a characteristic of injured tissues, such as areas of wound repair and solid tumor microenvironments^6,17^. The metabolic strategies enabling tumor cells to survive in acidic environments have been widely studied^4,17–20^, but little is known about the ability of human monocytes/macrophages to cope with these hostile conditions^15,16,52,53^. We show here that macrophages exposed to prolonged lactic acidosis strongly decrease their nutrient catabolism capacity, which leads to energy stress with mitochondrial depolarization and recourse to a transient phase of autophagy essential to their survival (Supplementary Fig. 11). We also report that the ketone body acetoacetate can fuel myeloid cells during acidosis, maintaining metabolic plasticity, energetic metabolism, and mitochondrial integrity and function (Supplementary Fig. 11). These results identify acetoacetate as a unique fuel to improve cell and tissue tolerance to acidic stress.

In conditions of extracellular acidosis, monocytes/macrophages enter a pseudostarvation state, displaying the biochemical and morphological characteristics of starving cells, despite the presence of nutrients and oxygen. Some tumor cells also respond to acidic microenvironments by entering a pseudostarvation phase^18,37^ associated with a large decrease in glucose uptake, glycolysis, and amino-acid consumption^18,20,52^. As in myeloid cells, extracellular acidification also resulted in a steep drop in pHi in tumor cells^18,54–56^. It is tempting to speculate that the decrease in pHi directly affects mitochondrial membrane potential. Moreover, a decrease in pHi also inhibits the activity of enzymes involved in glycolysis^43^ and acidosis-sensitive glutamine pumps^57,58^. This inhibition of the catabolic pathways responsible for supplying the TCA cycle and then the mitochondrial complexes with reducing equivalents (NADH, H^+^, FADH_2_) may also decrease the ability of the respiratory chain to maintain the mitochondrial membrane potential. Finally, a reduction of the pHi has been reported to trigger autophagy and mitophagy^18,54–56^. Sustained autophagy seems essential for tumor cell survival in acidic microenvironments^4^. Melanoma cells subjected to acidic stress display a pseudostarvation response and rely on autophagy to survive^18,29^. We also showed that non-proliferating human myeloid cells also transiently rely on an autophagic phase to survive in an acidic environment. Then, they gradually recovered at least part of their capacity to take up amino acids. Glutamine and leucine inhibit autophagy^59–61^, potentially explaining why extracellular acidosis-induced autophagy is transient and does not cause a metabolic crisis or cell death. However, LA-Mφ continued to be unable to metabolize glucose throughout the differentiation process, indicating that they did not recover their glycolytic capacity. Thus, while the autophagic phase is transient, metabolic stress is not, ending in a loss of metabolic plasticity in LA-Mφ. This limitation of cellular catabolic capacities might limit the energy production of LA-Mφ in response to stimulation.

There are various non-exclusive explanations for the inability of lactate oxidation to prevent pseudostarvation and energy stress in lactic acidosis. First, LDHA activity decreases with decreasing pHi^62^. Furthermore, lactate oxidation, which takes place in the cytosol and generates a proton, would not be expected to be favored in the event of a transient drop in pHi. Second, lactate oxidation generates pyruvate, which is then oxidized to generate one acetylCoA molecule, which can enter the TCA cycle provided that oxaloacetate is available^63^. Finally, lactate oxidation is coupled to the activity of the malate/aspartate shuttle and thus to the balance between malate, aspartate, and glutamate^63^, which is strongly disturbed by the decrease in amino-acid catabolism and the state of pseudo-starvation.

In contrast, in the presence of acetoacetate, LA-Mφ retains metabolic plasticity (Fig. 4). AcAc and β-OHB are the main ketone bodies present in the body. They serve as alternative fuels for the mitochondria of extrahepatic cells in cases of starvation or carbohydrate restriction^48^. The oxidation of AcAc into AcCoA is mediated by a CoA transferase (SCOT) that generates AcAc-CoA, followed by thiolases, yielding two molecules of AcCoA and one of succinate, both of which enter the TCA cycle (Supplementary Fig. 12)^48^. AcAc oxidation does not require ATP. AcAc oxidative flux occurs due to mass action: an abundant supply of AcAc and the rapid consumption of AcCoA via citrate synthase in the TCA cycle favors AcAc-CoA formation by OXCT1 (Supplementary Fig. 12)^48^. By contrast, hexokinase and acyl-CoA synthetases require ATP to generate and process AcCoA from glucose and fatty acids, respectively. This may explain why cells continue to be able to consume AcAc during lactic acidosis, whereas they are unable to metabolize glucose and amino acids. Finally, we observed that β-OHB did not protect macrophages from acidosis-induced pseudostarvation and mitochondrial membrane depolarization. One of the reasons may be that human monocyte, as murine macrophages^49^, poorly express BDH1 which oxidizes β-OHB into AcAc. Thus, AcAc appears to be a unique substrate able to protect myeloid cells from low extracellular pH-induced metabolic stress.

Importantly, even though AcAc prevents acidosis-induced pseudostarvation, mitochondrial depolarization, and autophagy, it does not prevent the drop of the pHi induced by lactic acidosis. We, therefore, suggest that AcAc, by feeding the TCA cycle and boosting mitochondrial respiration and biogenesis, prevents the depolarization of the mitochondrial membrane induced by the fall in pHi. In conclusion, our results suggest that AcAc protects cells from the metabolic stress induced by lactic acidosis (i) by providing them with fuel for the maintenance of their energy metabolism and (ii) by ensuring mitochondrial integrity and function. Nevertheless, we cannot rule out that the ability of AcAc to protect monocytes from lactic acid-induced mitochondrial depolarization and autophagy could be mediated, at least in part, through the signaling receptor GPR43 expressed by macrophages, as AcAc has been reported to signal via this short-chain fatty acid receptor^64,65^.

Many clinical situations in medicine are associated with tissue acidosis (areas of wound repair, solid tumor microenvironments) or systemic acidosis (septic shock). For instance, in sepsis, mitochondrial dysfunction (mitochondrial depolarization, reduction of mitochondrial respiration, and ATP production) occurs early, and its persistence contributes to organ failure and poor clinical prognosis^66–68^. The cause of the mitochondrial dysfunction in sepsis remains undetermined, but a role for inflammatory mediators has been suggested^69^. Sepsis is also associated with an increase in lactate production (with low levels of clearance by gluconeogenesis), which can lead to systemic acidosis (pH < 7.38) and is correlated with disease severity, morbidity, and mortality. Our findings on human myeloid cells chronically exposed to LA strongly suggest that acidosis is a major contributor to the mitochondrial dysfunction observed in situations in which extracellular acidosis occurs, such as sepsis.

Signs of cell starvation are observed during sepsis, together with a reduction of glucose, fatty acid, and amino acid catabolism, and hyperglycemia^69^. The observation that myeloid cells fail to take up nutrients during acidosis may, therefore, help to explain some of the metabolic changes associated with sepsis. Indeed, supplementation with glucose or nutrients is harmful in mouse models of bacterial sepsis, as such supplementation decreases host tolerance and increases host mortality^70^.

None of the treatments proposed for protecting mitochondria during sepsis are really efficient^71^. However, fasting and ketogenesis appear to be essential to animal survival and increase the ability of tissues to tolerate damage due to inflammation^70^. We show that AcAc protects mitochondria by enabling them to remain metabolically active in an acidic environment. As ketogenesis is affected in sepsis patients^72^, AcAc is potentially a candidate metabolite of choice for increasing cell tolerance to acidic stress, thereby preventing organ dysfunction and failure.

One limitation of this study is the use of a 5 mM AcAc concentration which, although similar to that used in many other in vitro studies, is elevated compared to physiological concentrations (20–150 µM in healthy adult subjects)^73,74^. This is presumably related to the fact that AcAc has a short half-life, thus requiring in vitro exposure of cells to a high concentration compared to physiological concentrations. The use of more stable forms would help to evaluate this hypothesis. Importantly, ketone bodies concentrations can increase dramatically during fasting or ketogenic diet, reaching concentrations (1–5 mM)^75^, similar to those used in this study. Moreover, although hepatocytes and gut epithelial cells abundantly express the mitochondrial isoform of HMGCS2, the key enzyme for ketone body synthesis^48^, studies report that other cell types, such as astrocytes^76^ and cancer-associated fibroblasts^77^, express HMGCS2, suggesting that extrahepatic ketogenesis may exist. Additional studies are required to investigate a potential local production of ketone bodies, especially in stressed tissues (exposed to acidosis and nutrient restriction).

In conclusion, we report that non-proliferating human monocytes/macrophages cope with an acidic microenvironment through a transient phase of autophagy and mitophagy, explaining their survival and ability to perform their functions in injured tissues and tumor lesions. We identify AcAc is a unique molecule that supports cellular metabolism during extracellular lactic acidosis, thereby maintaining mitochondrial integrity and preventing the need for a transient, but deleterious autophagic process. These results highlight the potential role of AcAc as a natural metabolite capable of protecting cells and enhancing host tolerance in pathological situations associated with acute or chronic acidosis.

## Methods

### Monocyte isolation and macrophage generation

Peripheral blood was obtained from anonymous healthy human volunteers (Blood collection center, Angers, France; agreement CPDL-PLER-2021 038 approved by the Ethics Committee of the University Hospital of Angers). In accordance with regulations, we only used blood from donors who have given their consent to use their blood for research studies. Peripheral blood mononuclear cells were isolated by standard density-gradient centrifugation on lymphocyte separation medium (Eurobio, Courtaboeuf, France). CD14^+^ monocytes were then isolated by positive magnetic cell-sorting (Miltenyi Biotec, Bergisch Gladbach, Germany). Monocytes (1 × 10^6^ cells/mL) were cultured in complete medium (CM) consisting of RPMI 1640 (Lonza, Verviers, Belgium) supplemented with 10% FCS (Eurobio), 2 mM L-glutamine, 1 mM sodium pyruvate, 0.1 mM non-essential amino acids, 10 mM HEPES, 100 U/mL penicillin and 100 µg/mL streptomycin (all from Lonza) in the presence of 50 ng/mL GM-CSF (R&D Systems, Minneapolis, MN). Experiments were performed in the presence of 10 mM lactic acid (Sigma-Aldrich, St Louis, MO) resulting in a pH of 6.5 to generate LA-Mφ (lactic acidosis conditioning), 10 mM HCl to achieve a final pH of 6.5 and to generate HCl-Mφ (acidosis conditioning), 10 mM sodium lactate (Sigma-Aldrich) to generate lactate-Mφ (lactosis conditioning, pH 7.3), in the presence or absence of 5 mM lithium acetoacetate (AcAc) (Sigma-Aldrich).

### Measurement of mitochondrial membrane potential

Mitochondrial membrane potential was assessed by incubating cells with 10 nM MitoTracker Green and 5 nM MitoTracker DeepRed (Thermo Fisher Scientific, Waltham, MA) in 1% bovine serum albumin (BSA) in PBS at 37 °C for 15 min. Cells were washed and incubated with 2 µg/mL 7-aminoactinomycin D (7-AAD) (Biolegend, San Diego, CA) to exclude dead cells. Flow cytometry data were acquired with a FACSCanto II flow cytometer (BD Biosciences, San Jose, CA) and analyzed with FlowJo software (Tree Star, Ashland, OR). In some experiments, LA-Mφ were sorted with flow cytometry using a FACS Aria cytometer (BD Biosciences) according to their mitochondrial polarization status. The purity of the sorted subpopulations was routinely >99%.

### Assessment of cell viability

The polarization of GM-Mφ and GM + LA-Mφ was initiated by adding bafilomycin or salinomycin (both from Sigma-Aldrich) to the culture medium at a concentration of 20 or 100 nM on day 2 and incubating for 24 h. Cell viability was then assessed by staining with 7-AAD before flow cytometry analysis. Cultures without bafilomycin or salinomycin were considered to be 100% viable.

### Measurement of intracellular pH

Intracellular pH (pHi) was measured by incubating 3 × 10^5^ Mφ with 500 nM pH-sensitive dye carboxy-SNARF-AM pH-sensitive dye (Life Technologies, Carlsbad, CA) in 1% BSA in PBS at 37 °C for 20 min and then performing flow cytometry analysis. We determined pHi as the ratio of fluorescence intensities at two emission wavelengths (585/42 nm and 700/60 nm PMTs). SNARF fluorescence in macrophages was calibrated with a high-potassium buffer (39.6 mM NaCl, 120 mM KCl, 2.3 mM CaCl_2_, 1 mM MgCl_2_, 5 mM HEPES, 10 mM glucose) at pH values of 6.5–8, in the presence of 10 µM nigericin (Sigma-Aldrich); nigericin exchanges external potassium with internal protons to equilibrate extracellular and intracellular pH.

### Analysis of mRNA levels

Cells were lysed in Trizol reagent (Life Technologies), and total RNA was extracted with the RNeasy Micro kit (Qiagen, Hilden, Germany) and then reverse-transcribed with the Superscript II reverse transcriptase (Life Technologies). Levels of mRNA encoding the indicated proteins were then analyzed by subjecting the cDNA obtained by reverse transcription to qPCR. Relative quantification was performed by the 2^−^^ΔΔCT^ method, normalized to housekeeping gene *RPS18*; results are expressed as relative mRNA levels. Primer sequences are listed in Supplementary Table 1.

### Oxygen consumption rate

We used an XF96 extracellular flux analyzer (Agilent Technologies, Santa Clara, CA) to determine the bioenergetic profile of intact cells. Day-4 cells were used to seed XF96 plates (50 × 10^3^ cells/well) and were allowed to recover for 24 h. Cells were then incubated in bicarbonate-free DMEM (Sigma-Aldrich) supplemented with 11 mM glucose, 2 mM l-glutamine, and 1 mM sodium pyruvate, in a CO_2_-free incubator for 1 h. Oxygen consumption rate (OCR) was recorded, to assess mitochondrial respiratory activity and glycolytic activity. OCR was recorded in basal conditions, and the cells were then treated sequentially with 2 µg/mL oligomycin, and 3 µM carbonyl cyanide p-(trifluoromethoxy) phenylhydrazone (FCCP) (both from Sigma-Aldrich). Non-mitochondrial respiration (OCR after treatment with 1 μg/mL antimycin A (Sigma-Aldrich)) was subtracted from all OCR measurements. ATP-linked respiration was estimated from the difference between the basal and oligomycin-inhibited respiration rates, and proton-leak respiration was obtained by subtracting non-mitochondrial respiration from the OCR measured after oligomycin treatment. Maximal respiratory capacity was determined as the rate of respiration in the presence of the uncoupler FCCP. Three independent replicates of each measurement were generated, and results were normalized according to cell concentration.

### Western blotting analysis

Levels of LC3-I, LC3-II, p62, β-actin, HSC-70, AMPKα, and pAMPKα were evaluated by western blotting. Cells were lysed in RIPA buffer containing protease inhibitors (Roche Applied Science, Penzberg, Germany). When indicated, cells were treated with 10 mM BafA1, 6 h before lysis. Lysates were centrifuged at 12,000 × *g* for 10 min at 4 °C to remove cell debris. Proteins (10 μg/lane) were separated by electrophoresis in a 4–20% polyacrylamide gel (Bio-Rad, Hercules, CA) in reducing conditions, and the resulting bands were transferred to a nitrocellulose membrane (Bio-Rad). Membranes were saturated in TBS/5% BSA/0.1% Tween 20 and then incubated for 16 h at 4 °C with polyclonal rabbit anti-LC3 (Cell Signaling Technology, Danvers, MA; ref 12741, 1/1000), anti-AMPKα (Cell Signaling Technology; ref 2532, 1/1000) or anti-phospho-AMPKα (Thr172) antibodies (Cell Signaling Technology; ref 2531, 1/1000) or with a mouse anti-p62 Ick ligand antibody (BD Biosciences, San Jose, CA; ref 610832, 1/1000). Protein loading was assessed by probing the membrane with a rabbit anti-β-actin (Abcam; ref ab227387, 1/5000) or a mouse anti-HSC-70 antibody (Santa Cruz; ref sc-7298, 1/2000). After washing, membranes were incubated with 1 μg/mL peroxidase-conjugated anti-rabbit or anti-mouse IgG antibody (Life Technologies), and bound antibodies were detected with the SuperSignal West Femto system (Thermo Fisher Scientific). Genetools software (version 4.01) from Syngene was used to quantify band intensity.

### Mitochondrial enzyme activities

The activities of complex IV of the respiratory chain and of citrate synthase were measured as described elsewhere^70^. Briefly, cell pellets (3 × 10^6^ cells) were resuspended in cell buffer (250 mM sucrose, 20 mM Tris, 2 mM EDTA, 1 mg/mL BSA, pH 7.4). They were subjected to a freeze-thaw cycle and then centrifuged (15,000 × *g*, 1 min). The pellet was resuspended in the same volume of cell buffer. For complex IV activity (cytochrome c oxidase), GM-Mφ and GM + LA-Mφ were resuspended in cell buffer at densities of 1 × 10^5^ and 2 × 10^5^ cells/mL, respectively, and 0.05 mM reduced cytochrome C, 1 mg/mL BSA and 0.25 mM laurylmaltoside were added to the reaction mixture. The rate of reduced cytochrome c oxidation was monitored at *λ* = 550 nm. Citrate synthase activity was measured as follows: the prewarmed reaction mixture (0.15 mM DTNB, 0.5 mM oxaloacetic acid, 0.3 mM acetyl CoA, 0.1% (v:v) Triton X100) was added to 2 × 10^5^ Mφ and the rate of appearance of CoA-SH was measured at *λ* = 412 nm.

### Metabolite quantification

The levels of glucose, glutamine, free l-amino acids, and lactate in cell culture supernatants and of acetyl-CoA and intracellular ATP in whole-cell lysates were determined with enzymatic assays, according to the manufacturer’s instructions (Abcam, Cambridge, UK). Results are expressed in µmol/10^6^ cells/24 h, with positive values indicating consumption and negative values indicating production. Acetoacetate concentrations were quantified with β-hydroxybutyrate dehydrogenase (3-HBDH) (Roche Applied Science), an enzyme that catalyzes the reversible reduction of acetoacetate to β-hydroxybutyrate in the presence of excess NADH, with the concomitant oxidation of NADH. The progress of the reaction was monitored by measuring the decrease in *A*_340nm_ due to the conversion of NADH into NAD. AcAc consumption was determined by subtracting the concentration of AcAc in cell culture supernatants from that in cell-free culture supernatants.

### Quantification of mtDNA copy number

Mitochondrial DNA (mtDNA) content was determined by qPCR with primers specific for the *ND4* and *COX1* genes and was weighted according to nuclear DNA levels, which were quantified by analyzing *B2M* and *GAPDH*. Primer sequences are listed in Supplementary Table 1.

### Macrophage stimulation

Day 5 Mφ, LA-Mφ, and LA-Mφ + AcAc were stimulated with 200 ng/mL LPS (Sigma-Aldrich). Cytokines were quantified by ELISA (R&D Systems) in the supernatants after 16 h. In some experiments, day 3 Mφ, LA-Mφ and LA-Mφ isolated according to their mitochondrial polarization status were either unstimulated or stimulated for 3 h with 200 ng/ml LPS before the analysis of *IL-6* and *TNFα* mRNA expression by RT-qPCR.

### Determination of relative cell size

Cell diameter was determined with the cell size analysis function of the Cellometer Auto T4 cell counter (Nexcelom Biosciences, Lawrence, MA); for each sample (*n* = 3), 150 individual cells were measured. Relative cell size was determined by flow cytometry with the FSC-A parameter and the exclusion of non-viable cells by 7-AAD staining. About 5000 individual cells were measured (*n* = 7).

### Confocal microscopy and image analysis

Cells were first incubated with 150 nM MitoTracker Green (Thermo Fisher Scientific) in CM medium at 37 °C for 10 min. The organization of the mitochondrial network was characterized by confocal microscopy and image analysis. Briefly, images were acquired on a Leica SP8 confocal microscope (Leica Microsystems, Nanterre, France) and submitted to a routine script in Matlab R2014a (The Mathworks, Natick, CA) for top-hat filtering (7 × 7 kernels), followed by median filtering (3 × 3 kernel) and binarization with the Otsu algorithm. Image blobs were then analyzed with the region props function of Matlab (MathWorks, Portola Valley, CA).

### Electron microscopy

We fixed 5 × 10^6^ macrophages by incubation for 16 h at 4 °C with 2.5% electron-grade glutaraldehyde (LFD Distribution, Sainte Consorce, France) in 0.1 M phosphate buffer pH 7.4. Samples were rinsed with 0.1 M phosphate buffer and post-fixed by incubation with 1% osmium tetroxide for 45 min at room temperature. Samples were then dehydrated in graded series of ethanol solutions and finally embedded in Epon at 60 °C for 48 h. Embedded samples were cut into 60 nm-thick sections, which were contrast-stained with 3% uranyl acetate in water for 10 min and then observed under a Jeol JEM 1400 transmission electron microscope operating at 120 keV and equipped with a Gatan Orius digital camera. We obtained full cross-sections of macrophages at high resolution by acquiring multiple fields at a magnification of 20,000 and stitching them together in Adobe Photoshop with the Photomerge routine. Stitched images were reviewed and scored by a trained electron histopathologist for the presence of autophagy and mitophagy vacuoles. The mitochondrial size was determined by measuring the maximum Feret diameter on TEM images.

### Statistical analysis

The results are presented with histograms (mean ± SD) or box and whisker plots (the boxplots display a median line, interquartile range boxes, min to max whiskers). Statistical analyses were performed with Graphpad PRISM version 7.02 (www.graphpad.com). The non-parametric two-tailed Mann–Whitney test and the two-way ANOVA followed by a Tukey post hoc test were used to compare two conditions or more, respectively. *P* values < 0.05 were considered statistically significant and *n* refers to different independent biological replicates.

### Reporting summary

Further information on research design is available in the Nature Research Reporting Summary linked to this article.

## Supplementary information

Supplementary Information

Reporting Summary

## Data Availability

The authors declare that the data supporting the findings of this study are available within the paper. Raw confocal and electron microscopy datasets have been deposited in the BioStudies database under accession code S-BSST716. Source data are provided with this paper. All other relevant data that support the findings of this study are available from the corresponding author upon reasonable request. Source data are provided with this paper.

## Source data

Source Data
