# Supplementary material for: Acetoacetate protects macrophages from lactic acidosis-induced mitochondrial dysfunction by metabolic reprograming
Source: Nat Commun. 2021 Dec 8;12:7115. doi: 10.1038/s41467-021-27426-x (PMC8655019; doi:10.1038/s41467-021-27426-x)
Supplement: Supplementary file 1 — Supplementary Information [file 41467_2021_27426_MOESM1_ESM.pdf]

Supplementary Material for:

Acetoacetate protects macrophages from lactic acidosis-induced mitochondrial dysfunction by metabolic reprograming

Clément Adam[1,†], Léa Paolini[1,†], Naïg Gueguen[2,3,†], Guillaume Mabilleau[4,5], Laurence Preisser[1], Simon Blanchard[1,6], Pascale Pignon[1], Florence Manero[7], Morgane Le Mao[2], Alain Morel[1,8], Pascal Reynier[9], Céline Beauvillain[1,6], Yves Delneste[1,6,‡], Vincent Procaccio[2,3,‡], Pascale Jeannin[1,6,‡,*]

[1] Univ Angers, Université de Nantes, INSERM, CRCINA, LabEx IGO, SFR ICAT, F-49000 Angers, France.

[2] Univ Angers, CHU d'Angers, INSERM, CNRS, MitoVasc, SFR ICAT, F-49000 Angers, France.

[3] Département de Biochimie et Génétique, CHU d'Angers, Angers, France.

[4] GEROM, Université d'Angers, Angers, France.

[5] Département de Pathologie cellulaire et tissulaire, CHU d'Angers, Angers, France.

[6] Laboratoire d'Immunologie et Allergologie, CHU d'Angers, Angers, France.

[7] Univ Angers, SFR ICAT, SCIAM, F-49000 Angers, France.

[8] Institut de Cancérologie de l'Ouest, F-49000 Angers, France.

[9] Laboratoire de Biochimie et biologie moléculaire, CHU d'Angers, Angers, France.

† These authors contributed equally to this work.

‡ These authors jointly supervised this work.

* Corresponding author : Pascale Jeannin: pascale.jeannin@univ-angers.fr

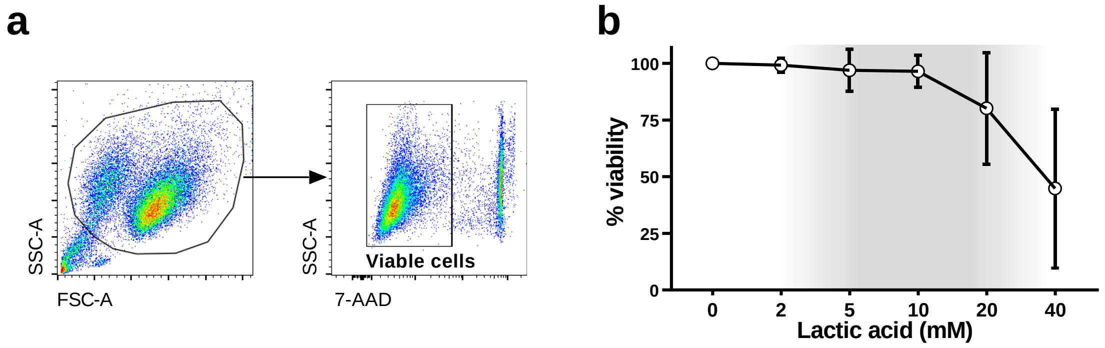

Supplementary figure 1. Macrophage viability in the presence of lactic acid. (a,b). Human monocytes were cultured with 50 ng/ml GM-CSF and the indicated concentrations of lactic acid. Cell viability was determined at day 5 by flow cytometry using 7-AAD staining. **(a)** gating strategy, **(b)** results are expressed as a percentage of viable cells normalized against non-treated condition for each donor (mean ± SD, n=5); shaded area, range of lactic acid concentrations in solid tumors (Sun, S. et al. Physiology (Bethesda) 32, 453–463 (2017)). Source data are provided in a Source Data file.

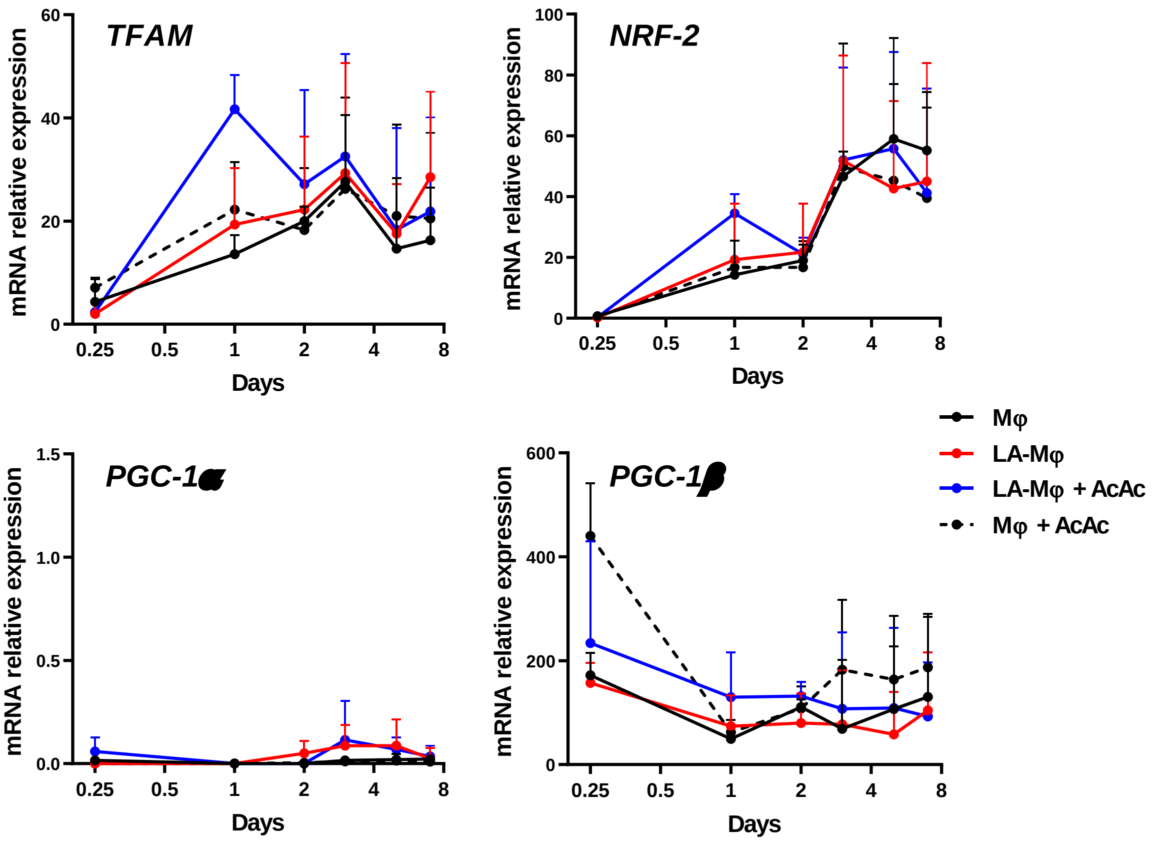

Supplementary figure 2. Mitochondrial biogenesis gene expression. Monocytes were differentiated into macrophages with 50 ng/ml GM-CSF in the absence (Mφ) or presence of 10 mM lactic acid (LA-Mφ), 5 mM acetoacetate (Mφ + AcAc) or both (LA-Mφ + AcAc). The levels of mRNA encoding *TFAM* (mitochondrial transcription factor A), *NRF-2* (nuclear respiratory factor 2), *PGC-1α* and *PGC-1β* (peroxisome proliferator-activated receptor gamma coactivator 1) were analyzed by RT-qPCR at the indicated time points and normalized to the housekeeping gene *RPS18* (mean ± SD, n=4-9). Source data are provided in a Source Data file.

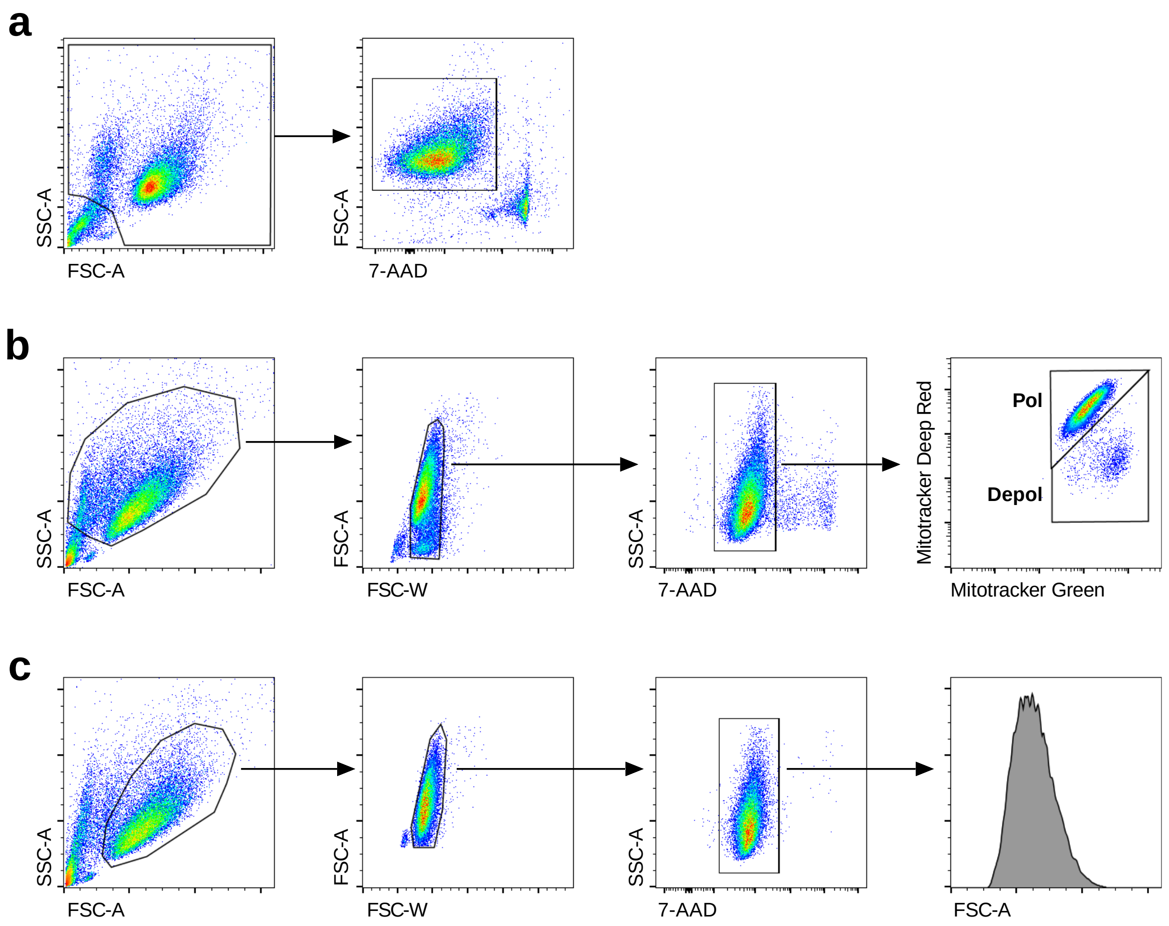

Supplementary figure 3. Gating strategies used for flow cytometry analysis. (a) Gating strategy to evaluate cell viability presented in Fig. 2i. **(b)** Gating strategy to measure the proportion of cells with depolarized mitochondrial membrane potential (Fig. 3a-b, supplementary Fig. 6, supplementary Fig. 8b, supplementary Fig. 9 and supplementary Fig. 10) and to sort "Pol" and "Depol" populations (Fig. 3c-d). **(c)** Gating strategy to estimate cell size presented in Fig. 3j.

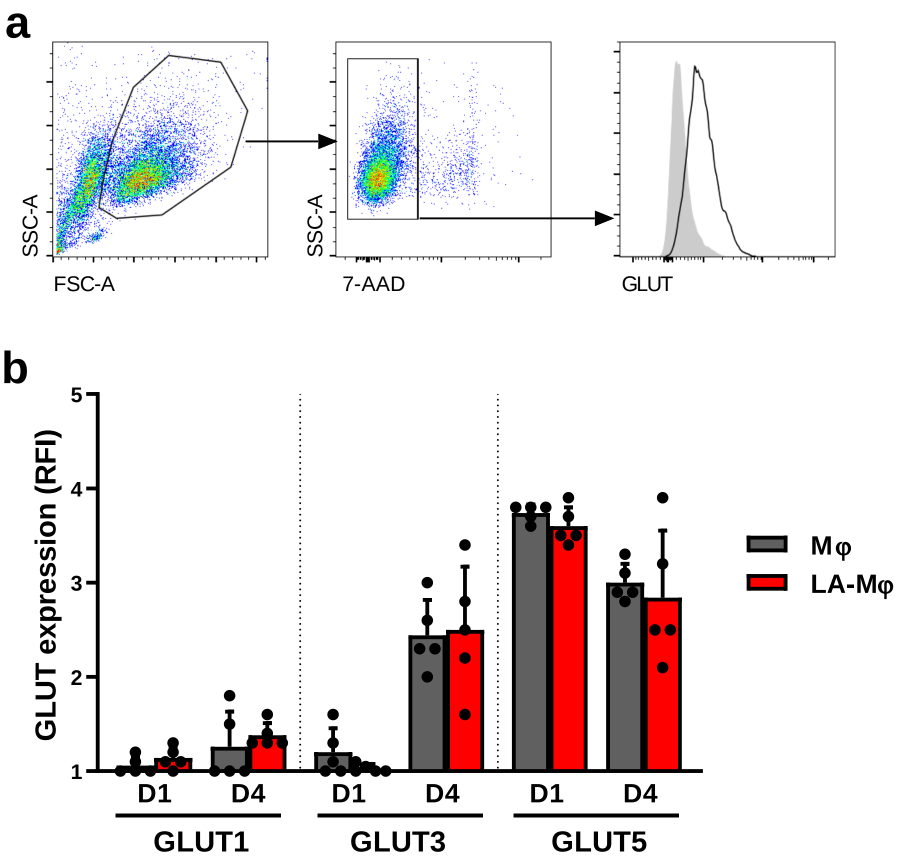

Supplementary figure 4. Lactic acid does not modulate GLUT expression by macrophages. (a,b). Monocytes were differentiated into macrophages with 50 ng/ml GM-CSF in the absence (Mφ) or presence of 10 mM lactic acid (LA-Mφ). The expression of GLUT1 (anti-GLUT1 APC antibody; R&D Systems, ref FAB1418A, 1/50), GLUT3 (anti-GLUT3 APC antibody; R&D Systems, ref FAB1415G, 1/20) and GLUT5 (anti-GLUT5 AF647 antibody; R&D Systems, ref FAB1349R, 1/100) was assessed by flow cytometry at day 1 and day 4. **(a)** Gating strategy, **(b)** results are expressed as RFI values (mean ± SD, n=5). Source data are provided in a Source Data file.

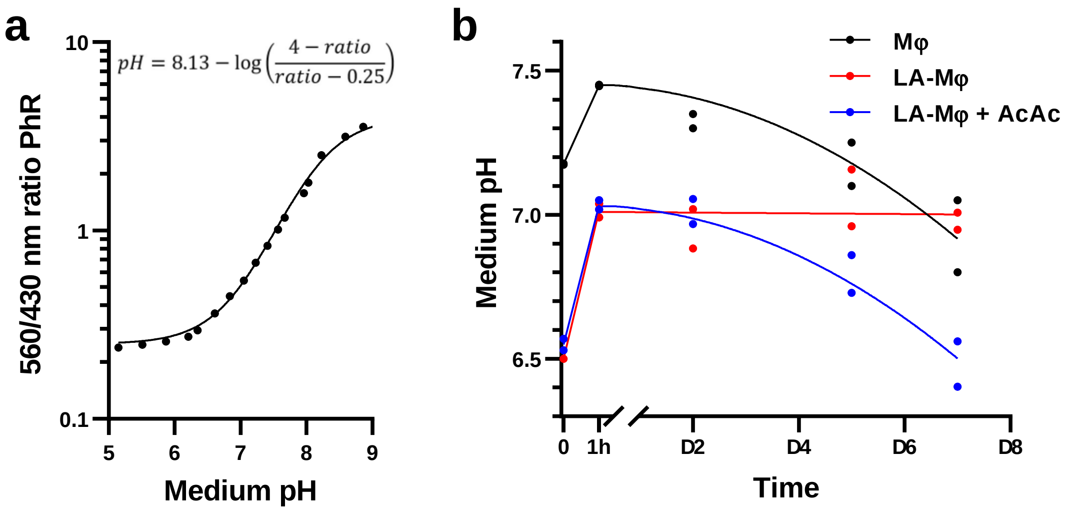

Supplementary figure 5. Monitoring culture medium pH. (a) To establish a standard curve, the culture medium was calibrated at the indicated pH in the absence of CO_2 with a pH-meter and the ratio of absorbance at 560 nm and 430 nm of phenol red (PhR) was determined by spectrophotometry (Michl et al, Commun. Biol. 2019, 2:144). The pH dependance of 560/430 nm absorbance ratio was fitted with the following curve: pH=8.13-log((4-ratio)/(ratio-0.25)). **(b)** Monocytes were differentiated into macrophages in the absence (Mφ) or presence of 10 mM LA (LA-Mφ), without or with 5 mM AcAc (LA- Mφ + AcAc). The pH of the medium was evaluated at the indicated time points by monitoring the ratio of absorbances at 560 nm/430 nm. Data from 2 independent experiments are shown. Source data are provided in a Source Data file.

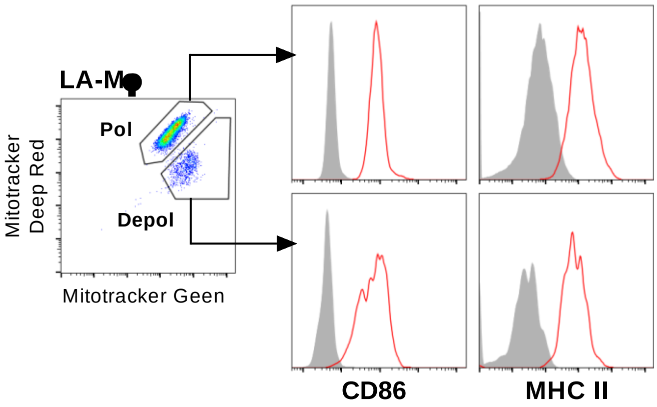

Supplementary figure 6. CD86 and MHC II expression on day 3 LA-Mφ without and with depolarized mitochondria. Monocytes were differentiated into Mφ for 3 days with 50 ng/ml GM-CSF in the presence of 10 mM lactic acid (LA-Mφ). Mitochondrial membrane potential (ΔΨm) was analyzed by flow cytometry using Mitotracker Green and Mitotracker Deep Red probes and the expression of CD86 (anti-CD86 BV421 antibody, BD Biosciences, ref 562433, 1/100) and MHC II (anti-HLA-DR V450 antibody, BD Biosciences, ref 561359, 1/50) was evaluated on cells without (Pol) or with (Depol) depolarized mitochondria. Representative dot plots and histograms of one of three independent experiments are shown. Gating strategy is shown in supplementary Fig. 3b.

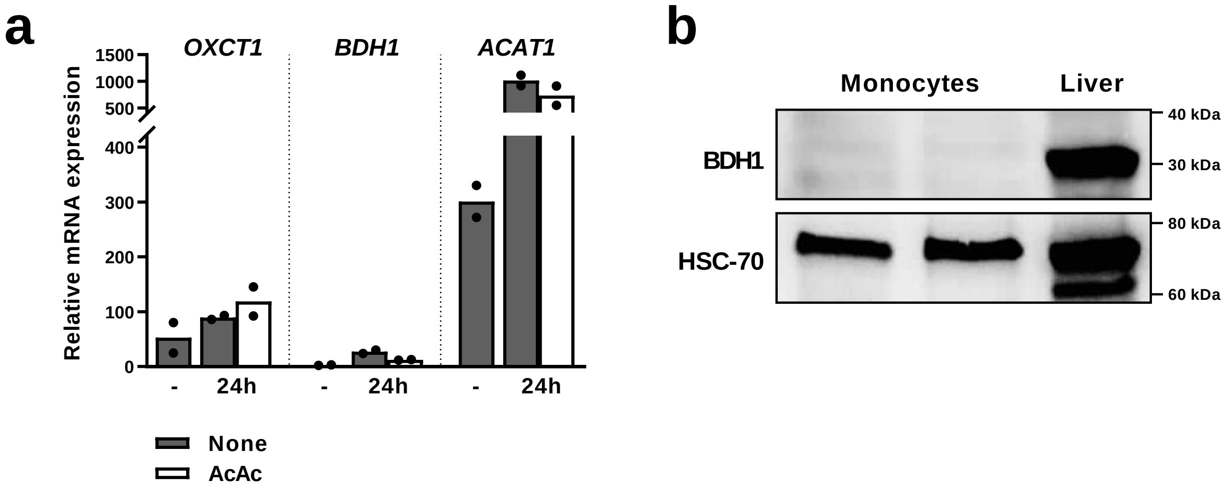

Supplementary figure 7. BDH1 is not expressed by human monocytes. (a) mRNA expression of *OXCT1*, *BDH1* and *ACAT1* was assessed by RT-qPCR in freshly isolated monocytes and in monocytes cultured for 24 hours in the presence of GM-CSF, without or with 5 mM AcAc. Data from 2 independent experiments are shown. **(b)** Western blotting analysis of BDH1 (anti-BDH1 Ab; Proteintech, Rosemont, IL; ref 15417-1-AP, 1/1000) and HSC-70 (anti-HSC-70; Santa Cruz; ref sc7298, 1/2000) in protein lysates (20 µg/lane) from freshly purified monocytes (first two lanes, isolated from two different donors). Total liver protein extract (Biochain, Newark, CA; ref P1234149) was used as a positive control for BDH1 expression. Representative results from three independent experiments. Source data are provided in a Source Data file.

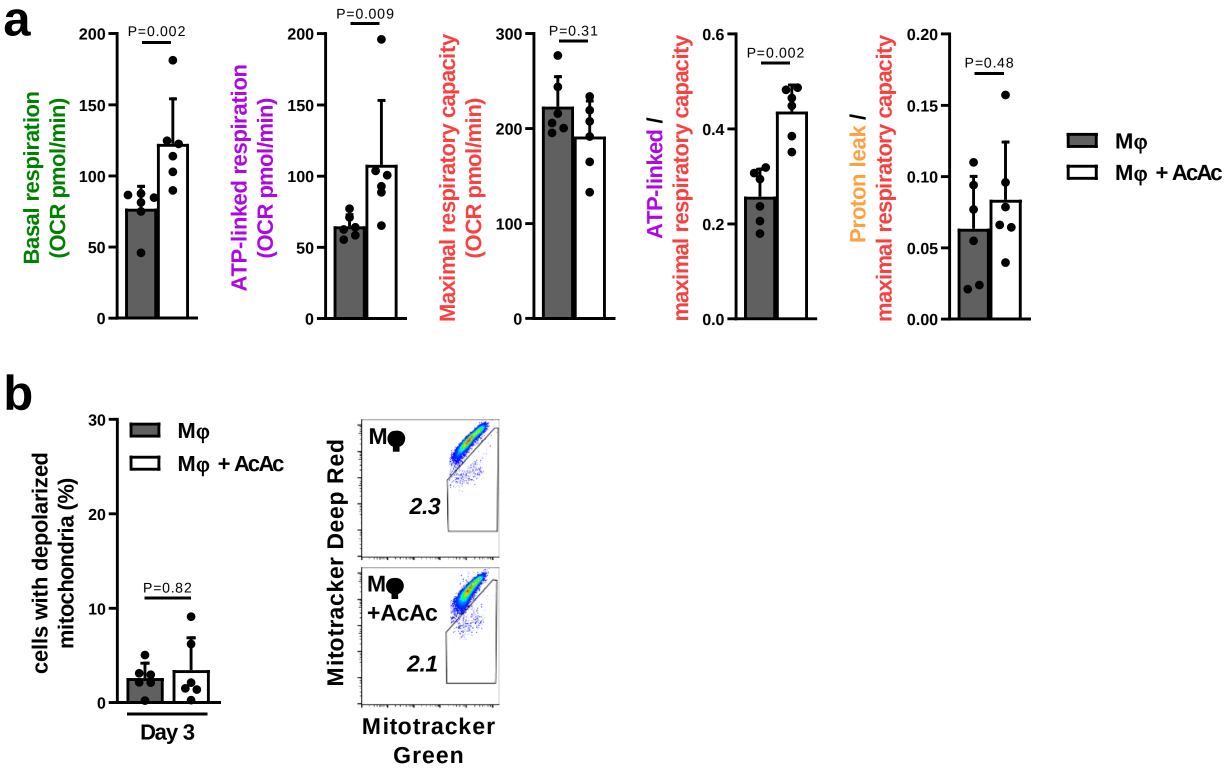

Supplementary figure 8. Impact of AcAc on mitochondrial respiration. (a) Monocytes were differentiated into macrophages with GM-CSF in the absence (Mφ) or presence of acetoacetate (Mφ+AcAc). Metabolic parameters were obtained from oxygen consumption rate (OCR) monitored at day 4 (mean ± SD; n=6). **(b)** Cells with depolarized mitochondrial membrane potential (ΔΨm) were analyzed by flow cytometry using Mitotracker Green and Mitotracker Deep Red probes at day 3. Values correspond to the percentage of cells with depolarized mitochondria (mean ± SD; n=6). Representative dot plots are shown. Gating strategy is shown in supplementary Fig. 3b. Two-tailed Mann-Whitney U test was performed for statistical analysis. Source data are provided in a Source Data file.

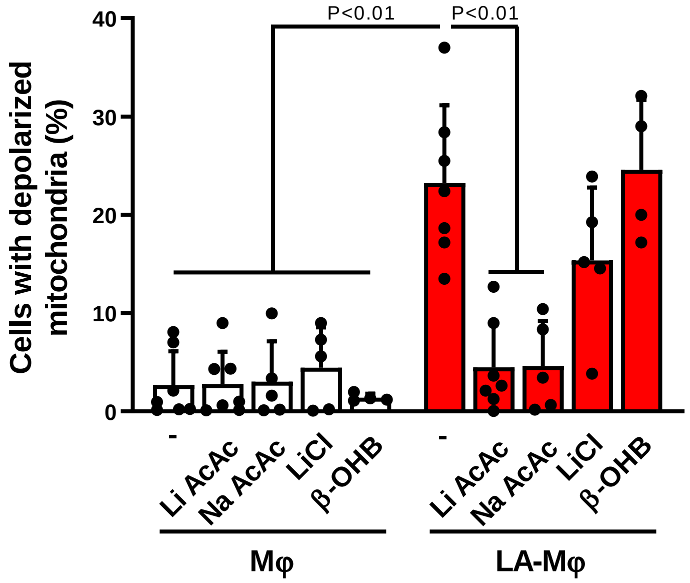

Supplementary figure 9. Effects of acetoacetate on mitochondrial depolarization induced by lactic acid. Monocytes were differentiated into macrophages with 50 ng/ml GM-CSF in the absence (Mφ) or presence of 10 mM lactic acid (LA-Mφ), 5 mM lithium acetoacetate (Li AcAc), 5 mM sodium acetoacetate (Na AcAc, MedChemExpress, Monmouth Junction, NJ, ref HY-112540B), lithium chloride (LiCl) or β-hydroxybutyrate (β-OHB). Cells with depolarized mitochondrial membrane potential (ΔΨm) were analyzed by flow cytometry using Mitotracker Green and Mitotracker DeepRed probes at day 3. Results are expressed as a percentage of cells with depolarized mitochondria (Mean ± SD, n=7 for the control and Li AcAc conditions; n=5 for the Na AcAc and LiCl conditions; n=4 for the β-OHB condition). Gating strategy is shown in supplementary Fig. 3b. Two-tailed Mann-Whitney U test was performed for statistical analysis. Source data are provided in a Source Data file.

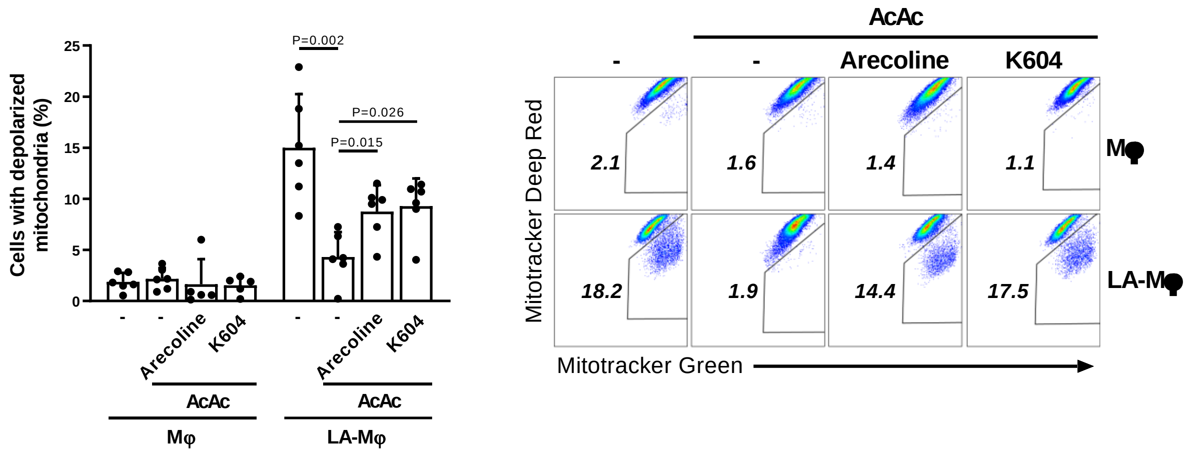

Supplementary figure 10. AcAc prevents LA-induced mitochondrial depolarization through ACAT1. Monocytes were differentiated into macrophages with 50 ng/ml GM-CSF in the absence (Mφ) or presence of 10 mM lactic acid (LA-Mφ), without or with 5 mM acetoacetate (AcAc). In some experiments, the ACAT1 inhibitors, arecoline hydrobromide (250 µM) or K604 (20 µM) (both from Sigma Aldrich, St Louis, MO; ref 31593 and ref SML1837, respectively) were added during the differentiation. Cells with depolarized mitochondrial membrane potential (ΔΨm) were analyzed at day 3 by flow cytometry using Mitotracker Green and Mitotracker DeepRed probes. Values correspond to the percentage of cells with depolarized mitochondria (Mean ± SD, n=6). Two-tailed Mann-Whitney U test was performed for statistical analysis. Representative dot plots are shown. Gating strategy is shown in supplementary Fig. 3b. Source data are provided in a Source Data file.

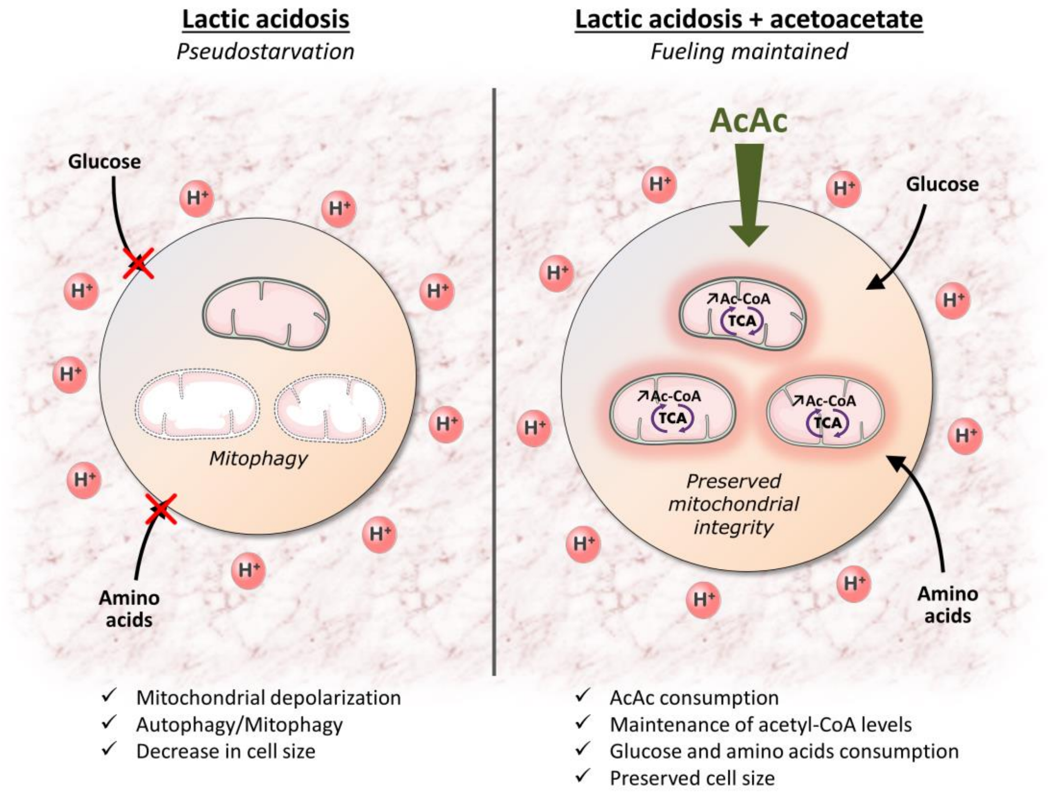

Supplementary figure 11. Schematic representation of the protective role of AcAc on macrophages under prolonged lactic acidosis. High levels of cell proliferation and/or tissue hypoxia lead to the extracellular accumulation of LA and acidification. Macrophages are then subjected to metabolic stress (they stop consuming nutrients, their cell size/diameter and intracellular levels of AcCoA decrease) and display mitochondrial dysfunction (mitochondrial membrane depolarization, decrease in mitochondrial mass), but they survive through autophagy. AcAc acts as a unique fuel that protects mitochondria of myeloid cells exposed to acidosis, thereby preventing pseudostarvation and the need for autophagy to survive. Abbreviations: AcAc, acetoacetate; Ac-CoA, acetyl coenzyme A; TCA, tricarboxylic acid cycle; H^+, proton.

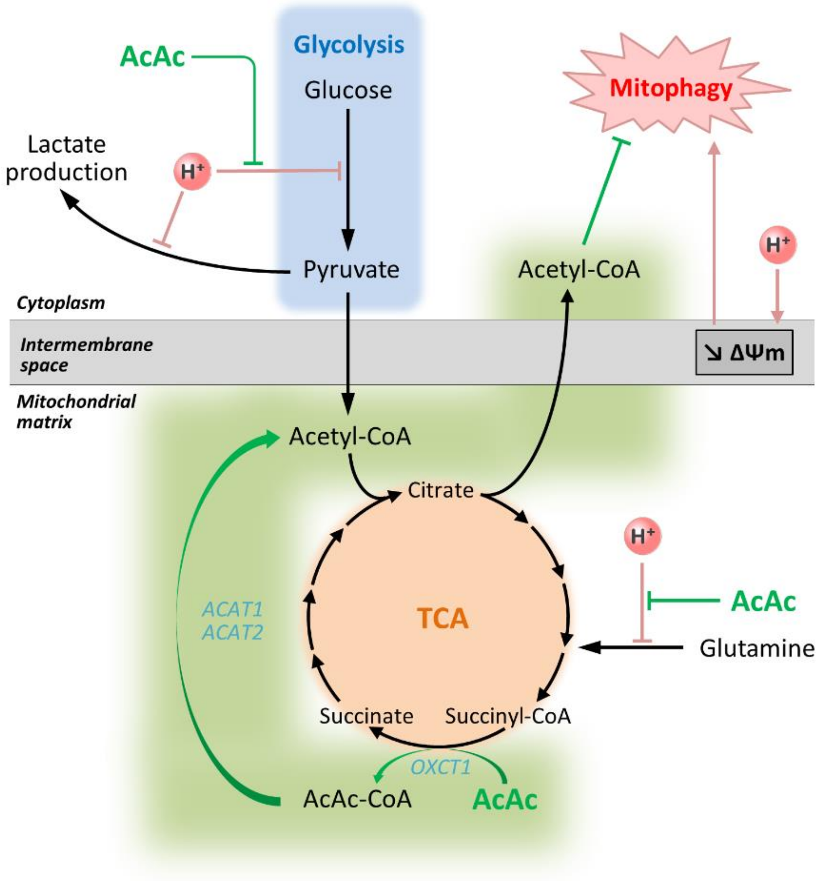

Supplementary figure 12. Schematic representation of the impact of AcAc on the macrophage metabolism during acidosis. Human macrophages subjected to lactic acidosis display a large decrease in nutrient uptake, low levels of intracellular acetyl-CoA, and a depolarization of mitochondria, rendering them dependent on autophagy to survive. AcAc (which is assimilated into AcAc-CoA via OXCT1 and acetyl-CoA via ACAT1 and ACAT2) can serve as an alternative fuel for macrophages exposed to lactic acidosis. AcAc maintains the ability of these cells to take up glucose and amino acids, along with normal levels of acetyl-CoA, thereby ensuring an adequate supply of substrates, maintaining mitochondrial function and integrity, and preventing starvation-induced autophagy. Abbreviations: AcAc, acetoacetate; CoA, coenzyme A; TCA, tricarboxylic acid cycle; H^+, proton; Δψm, membrane potential; ACAT1/2, Acetyl-CoA acetyltransferase 1/2; OXCT1, 3-oxoacid CoA-transferase 1.

Supplementary Table 1: Primer list

**Primer name**	**sequence**
ND4	F : GCTAGTCATATTAAGTTGTTG<br>R : CGCACTAATTTACACTCA
COX1	F : TCCACTATGTCCTATCAATA<br>R : GGTGTAGCCTGAGAATAG
B2M	F : CAGCTCTAACATGATAACC<br>R : CCTGTAGGATTCTTCTTTC
GAPDH	F : CCCTGTCCAGTTAATTTC<br>R : CACCCTTTAGGGAGAAAA
ATG5	F : CAGTTTTGGGCCATCAATCG<br>R : CCTAGTGTGTGCAACTGTCC
p62	F : GAATCAGCTTCTGGTCCATCG<br>R : GAACTCTCTGGAGAGACGGG
LC3B	F : ACCCTGAGTCTTCTCTTCAGG<br>R : CAACTGTGATGGCAAATGCG
HMOX1	F : CTCAACATCCAGCTCTTTGAGG<br>R : CTGAGTGTAAGGACCCATCG
BNIP3L1	F : CTTCGCCACAAGAAGATGGG<br>R : CTGAAGTGGAACTCCTTGGG
TNFα	F : CACTTTGGAGTGATCGGCCCC<br>R : GCTGGTTATCTCTCAGCTCCACG
IL-6	F : CTGGCAGAAAACAACCTGAAC<br>R : CAGGGGTGGTTATTGCATCTA
TFAM	F : GATTCACCGCAGGAAAAGC<br>R : TTGTGCGACGTAGAAGATCC
NRF-2	F : AGCACATCCAGTCAGAAACC<br>R : AAGTGACTGAAACGTAGCCG
PGC-1α	F : TCTGACCACAAACGATGACC<br>R : GAACCCTTGGGGTCATTTGG
PGC-1β	F : AAAAGGCCATTGGGGAAGG<br>R : TGATGAAGCCGTACTTCTCG
