# Supplementary material for: Acetoacetate protects macrophages from lactic acidosis-induced mitochondrial dysfunction by metabolic reprograming
Source: Nat Commun. 2021 Dec 8;12:7115. doi: 10.1038/s41467-021-27426-x (PMC8655019; doi:10.1038/s41467-021-27426-x)
Supplement: Supplementary file 2 — Reporting Summary [file 41467_2021_27426_MOESM2_ESM.pdf]

nature research

Corresponding author(s): Pascale JEANNIN

Last updated by author(s): Sep 24, 2021

Reporting Summary

Nature Research wishes to improve the reproducibility of the work that we publish. This form provides structure for consistency and transparency in reporting. For further information on Nature Research policies, see our Editorial Policies and the Editorial Policy Checklist.

Statistics

For all statistical analyses, confirm that the following items are present in the figure legend, table legend, main text, or Methods section.

n/a	Confirmed	
☐	☒	The exact sample size (*n*) for each experimental group/condition, given as a discrete number and unit of measurement
☐	☒	A statement on whether measurements were taken from distinct samples or whether the same sample was measured repeatedly
☐	☒	The statistical test(s) used AND whether they are one- or two-sided<br>*Only common tests should be described solely by name; describe more complex techniques in the Methods section.*
☒	☐	A description of all covariates tested
☒	☐	A description of any assumptions or corrections, such as tests of normality and adjustment for multiple comparisons
☐	☒	A full description of the statistical parameters including central tendency (e.g. means) or other basic estimates (e.g. regression coefficient) AND variation (e.g. standard deviation) or associated estimates of uncertainty (e.g. confidence intervals)
☐	☒	For null hypothesis testing, the test statistic (e.g. *F*, *t*, *r*) with confidence intervals, effect sizes, degrees of freedom and *P* value noted<br>*Give P values as exact values whenever suitable.*
☒	☐	For Bayesian analysis, information on the choice of priors and Markov chain Monte Carlo settings
☒	☐	For hierarchical and complex designs, identification of the appropriate level for tests and full reporting of outcomes
☒	☐	Estimates of effect sizes (e.g. Cohen's *d*, Pearson's *r*), indicating how they were calculated

Our web collection on statistics for biologists contains articles on many of the points above.

Software and code

Policy information about availability of computer code

Data collection	no software was used for data collection
Data analysis	GraphPad Prism v7.02, FlowJo v10, Genetools v4.01, Adobe Photoshop CS2 v9.0.2

For manuscripts utilizing custom algorithms or software that are central to the research but not yet described in published literature, software must be made available to editors and reviewers. We strongly encourage code deposition in a community repository (e.g. GitHub). See the Nature Research guidelines for submitting code & software for further information.

Data

Policy information about availability of data

All manuscripts must include a data availability statement. This statement should provide the following information, where applicable:

- Accession codes, unique identifiers, or web links for publicly available datasets
- A list of figures that have associated raw data
- A description of any restrictions on data availability

The authors declare that the data supporting the findings of this study are available within the paper. Raw confocal and electron microscopy datasets have been deposited in the BioStudies database under accession code S-BSST716 [https://www.ebi.ac.uk/biostudies//studies/S-BSST716]. Source data are provided with this paper.

Field-specific reporting

Please select the one below that is the best fit for your research. If you are not sure, read the appropriate sections before making your selection.

☒ Life sciences ☐ Behavioural & social sciences ☐ Ecological, evolutionary & environmental sciences

For a reference copy of the document with all sections, see nature.com/documents/nr-reporting-summary-flat.pdf

Life sciences study design

All studies must disclose on these points even when the disclosure is negative.

Sample size	Sample sizes were not predetermined by statistical methods. Sample sizes were chosen based on our prior experience and in order to be able to carry out statistical tests, and were similar to those generally employed in the field. All sample sizes are shown in the figure legends of our manuscript.
Data exclusions	No data were excluded from the analysis
Replication	All experiments were independently replicated at least 2-3 times and were successful. Number of repeats is provided in the figure legends.
Randomization	Blood samples from which primary monocytes were purified were obtained from randomized healthy donors from the blood collection center of the university hospital of Angers, France.
Blinding	Stitched images from TEM were reviewed and scored by a trained electron histopathologist in a blinded manner. For the other samples, blinding was not relevant as none of the readouts were subjective.

Reporting for specific materials, systems and methods

We require information from authors about some types of materials, experimental systems and methods used in many studies. Here, indicate whether each material, system or method listed is relevant to your study. If you are not sure if a list item applies to your research, read the appropriate section before selecting a response.

Materials & experimental systems

n/a	Involved in the study	
☐	☒	Antibodies
☒	☐	Eukaryotic cell lines
☒	☐	Palaeontology and archaeology
☒	☐	Animals and other organisms
☒	☐	Human research participants
☒	☐	Clinical data
☒	☐	Dual use research of concern

Methods

n/a	Involved in the study	
☒	☐	ChIP-seq
☐	☒	Flow cytometry
☒	☐	MRI-based neuroimaging

Antibodies

Antibodies used

1. mAb rabbit anti-human LC3A/B (clone D3U4C), Cell Signaling Technology, Cat#12741 (1:1000 dilution)
2. pAb rabbit anti-human AMPK alpha, Cell Signaling Technology, Cat#2532 (1:1000 dilution)
3. pAb rabbit anti-human phospho-AMPK alpha (Thr172), Cell Signaling Technology, Cat#2531 (1:1000 dilution)
4. mAb mouse anti-human p62 lck ligand (clone 3/P62 LCK LIGAND), BD Biosciences, Cat#610832 (1/1000 dilution)
5. pAb rabbit anti-human beta-actin, Abcam, Cat#ab227387 (1/5000 dilution)
6. mAb mouse anti-human HSC-70 (clone B-6), Santa Cruz, Cat#sc-7298 (1/2000 dilution)
7. pAb goat anti-rabbit IgG HRP, Invitrogen, Cat#G-21234 (1/1000 dilution)
8. pAb goat anti-mouse IgG HRP, Invitrogen, Cat#G-21040 (1/1000 dilution)
9. mAb mouse anti-human GLUT1 APC (clone 202915), R&D Systems, Cat#FAB1418A (1/50 dilution)
10. mAb mouse anti-human GLUT3 APC (clone 202017), R&D Systems, Cat#FAB1415G (1/20 dilution)
11. mAb mouse anti-human GLUT5 AF647 (clone 195205), R&D Systems, Cat#FAB1349R (1/100 dilution)
12. mAb mouse anti-human CD86 BV421 (clone 2331), BD Biosciences, Cat#562433 (1/100 dilution)
13. mAb mouse anti-human HLA-DR V450 (clone G46-6), BD Biosciences, Cat#561359 (1/50 dilution)
14. pAb rabbit anti-human BDH1, Proteintech, Cat#15417-1-AP (1/1000 dilution)

Validation

All antibodies are commercially available, and validated by manufacturer.
1. https://www.cellsignal.com/products/primary-antibodies/lc3a-b-d3u4c-xp-rabbit-mab/12741
2. https://www.cellsignal.com/products/primary-antibodies/ampka-antibody/2532
3. https://www.cellsignal.com/products/primary-antibodies/phospho-ampka-thr172-antibody/2531
4. https://www.bdbiosciences.com/en-us/products/reagents/microscopy-imaging-reagents/immunofluorescence-reagents/purified-

mouse-anti-p62-ick-ligand.610832
5. https://www.abcam.com/beta-actin-antibody-ab227387.html
6. https://www.scbt.com/fr/p/hsc-70-antibody-b-6
7. https://www.thermofisher.com/antibody/product/Goat-anti-Rabbit-IgG-H-L-Cross-Adsorbed-Secondary-Antibody-Polyclonal/G-21234
8. https://www.thermofisher.com/antibody/product/Goat-anti-Mouse-IgG-H-L-Cross-Adsorbed-Secondary-Antibody-Polyclonal/G-21040
9. https://www.rndsystems.com/products/human-glut1-apc-conjugated-antibody-202915_fab1418a
10. https://www.rndsystems.com/products/human-glut3-alexa-fluor-488-conjugated-antibody-202017_fab1415g
11. https://www.rndsystems.com/products/human-glut5-alexa-fluor-647-conjugated-antibody-195205_fab1349r
12. https://www.bdbiosciences.com/en-us/products/reagents/flow-cytometry-reagents/research-reagents/single-color-antibodies-ruo/bv421-mouse-anti-human-cd86.562433
13. https://www.bdbiosciences.com/en-us/products/reagents/flow-cytometry-reagents/research-reagents/single-color-antibodies-ruo/v450-mouse-anti-human-hla-dr.561359
14. https://www.ptglab.com/products/BDH1-Antibody-15417-1-AP.htm

Flow Cytometry

Plots

Confirm that:

- [x] The axis labels state the marker and fluorochrome used (e.g. CD4-FITC).
- [x] The axis scales are clearly visible. Include numbers along axes only for bottom left plot of group (a 'group' is an analysis of identical markers).
- [x] All plots are contour plots with outliers or pseudocolor plots.
- [x] A numerical value for number of cells or percentage (with statistics) is provided.

Methodology

Sample preparation	For macrophages preparation, peripheral blood mononuclear cells (PBMC) were obtained from healthy donors, monocytes were purified by magnetic cell sorting and differenciated for 5 days (or otherwise specified) in the presence of GM-CSF and the different components studied.<br>For flow cytometry cell surface staining, 200 000 cells/100µl were incubated with non specific human IgG before staining with fluorochrome-conjugated antibodies.<br>MitoTracker, SNARF or 7-AAD staining were performed according to the manufacturer's instructions.
Instrument	FACSCanto II
Software	FACS Diva
Cell population abundance	The purity of the sorted macrophages according to their mitochondrial polarization status (Mitotracker labelling) was routinely >99% and was checked by re-acquisition of a part of the sorted population.
Gating strategy	gating on FSC/SSC + viability marker (doublet discrimination was performed for cell sorting)

- [x] Tick this box to confirm that a figure exemplifying the gating strategy is provided in the Supplementary Information.
